# Pd-Catalyzed Suzuki-Miyaura Cross-Coupling of Pentafluorophenyl Esters

**DOI:** 10.3390/molecules23123134

**Published:** 2018-11-29

**Authors:** Jonathan Buchspies, Daniel J. Pyle, Huixin He, Michal Szostak

**Affiliations:** Department of Chemistry, Rutgers University, 73 Warren Street, Newark, NJ 07102, USA; jonathan.buchspies@gmail.com (J.B.); danielj.pyle@gmail.com (D.J.P.)

**Keywords:** Suzuki-Miyaura, cross-coupling, aryl esters, C–O activation, Pd-catalysis

## Abstract

Although the palladium-catalyzed Suzuki-Miyaura cross-coupling of aryl esters has received significant attention, there is a lack of methods that utilize cheap and readily accessible Pd-phosphane catalysts, and can be routinely carried out with high cross-coupling selectivity. Herein, we report the first general method for the cross-coupling of pentafluorophenyl esters (pentafluorophenyl = pfp) by selective C–O acyl cleavage. The reaction proceeds efficiently using Pd(0)/phosphane catalyst systems. The unique characteristics of pentafluorophenyl esters are reflected in the fully selective cross-coupling vs. phenolic esters. Of broad synthetic interest, this report establishes pentafluorophenyl esters as new, highly reactive, bench-stable, economical, ester-based, electrophilic acylative reagents via acyl-metal intermediates. Mechanistic studies strongly support a unified reactivity scale of acyl electrophiles by C(O)–X (X = N, O) activation. The reactivity of pfp esters can be correlated with barriers to isomerization around the C(acyl)–O bond.

## 1. Introduction

The recent emergence of Suzuki-Miyaura cross-coupling of amide and ester electrophiles by selective C(acyl)–X cleavage represents one of the most promising approaches to functionalization of the traditionally inert amide and ester bonds in organic synthesis [1,2,3]. Although a broad range of amide precursors have been explored [4,5,6,7,8], N.B. benefiting from amide twist [9,10,11,12,13], Pd-catalyzed cross-coupling of esters has received significantly less attention. The seminal study by Newman in 2017 reported the [Pd(NHC)(cin)Cl]-catalyzed cross-coupling of aryl esters at high temperature [14]. Subsequently, we have reported a general method for the cross-coupling of both esters and amides at room temperature [15]. Further studies established that various Pd(II)-NHC precatalysts are significantly more reactive after optimizing conditions [16,17]. Moreover, Hazari demonstrated the cross-coupling of aryl esters at room temperature conditions using strong bases [18].

This strategy to develop cross-coupling reactions of aryl esters hinges upon ground-state destabilization of the barrier to rotation around the C(acyl)–O bond [19]. In contrast to amides, esters feature significant stabilization in the transition state. Given the established capacity of pentafluorophenyl esters as acyl transfer reagents in nucleophilic addition reactions [20,21,22], we recently questioned whether the ground-state-destabilization principle might enable facile cross-coupling of pentafluorophenyl esters under chemoselective conditions that are inaccessible to the current-state-of-the-art phenolic esters [1,2,3]. In this *Special Issue on Amide Bond Activation*, we report the successful realization of this approach, and report the first general method for the cross-coupling of pentafluorophenyl esters by selective C–O acyl cleavage. Notable features of our findings include: (1) The first Pd-phosphane-catalyzed Suzuki cross-coupling of esters by C–O activation; (2) unprecedented selectivity of the cross-coupling; (3) establishment of the reactivity scale in the cross-coupling of bench-stable acyl electrophiles catalyzed by Pd-phosphanes.

**Scheme 1:**
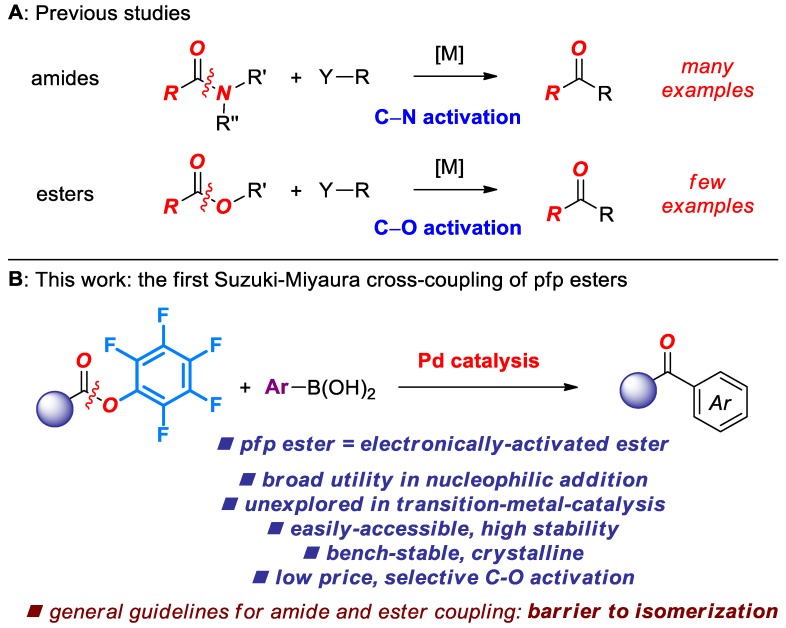
Cross-coupling of amides and esters by C–N and C–O activation.

Notably, this study establishes pentafluorophenyl esters as new, highly reactive, bench-stable, economical, ester-based, electrophilic acylative reagents via acyl-metals [1,2,3]. Considering the versatile role of pfp esters in organic synthesis [20,21,22], we expect that this approach will find wide application in the development of cross-coupling reactions of bench-stable ester electrophiles by acyl [1] and decarbonylative pathways [2,7,23,24] (Scheme 1). 

## 2. Results

Cross-coupling of pentafluorophenyl benzoate with 4-tolyl boronic acid was selected as our model system. From the outset we sought to develop a catalytic system based on phosphane ligands, due to the low price, ready availability and orthogonal selectivity compared to the more σ-donating NHCs. Selected optimization results are presented in Table 1. As expected, the choice of base (entries 1–6), phosphane ligand (entries 7–12), palladium catalyst (entries 13–15), palladium to ligand ratio (entries 16–18), stoichiometry (entries 19–20) and concentration (entries 21–22) had a major impact on the cross-coupling efficiency. Finally, we established that the optimum conditions involved using Pd_2_(dba)_3_ (3.0 mol%) as a catalyst, PCy_3_HBF_4_ (12 mol%) as a ligand, and Na_2_CO_3_ (4.5 equiv) as a base in dioxane at 120 °C (entry 20). Importantly, under the optimized conditions cleavage of the alternative O–C(Ar) bond or nucleophilic addition to the activated pfp group were not observed. To our knowledge, the reaction represents the first example of a Pd-phosphane-catalyzed Suzuki cross-coupling of an ester group by selective C–O cleavage [1,2,3,23,24]. It should be noted that all reaction components are easy-to-handle bench-stable solids, which represents a significant practical advantage over related cross-coupling protocols. 

**Figure 1:**
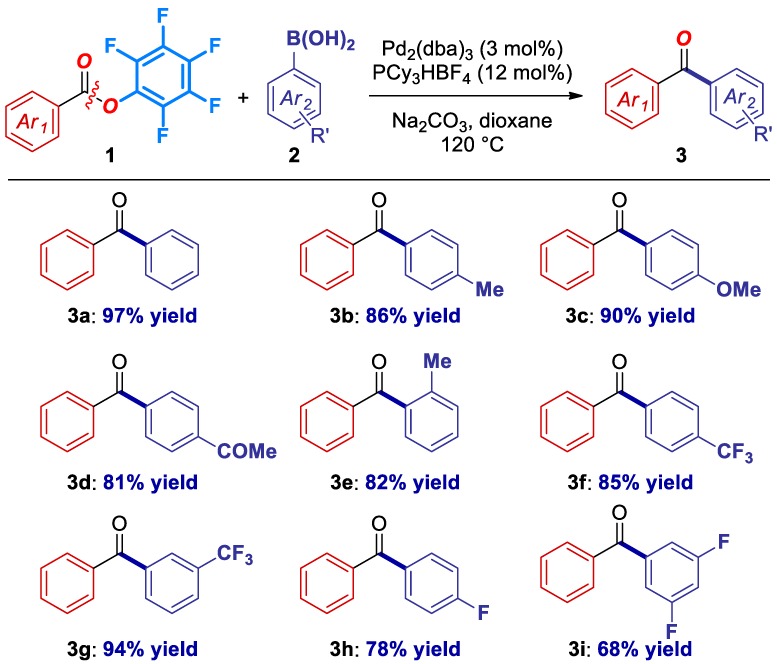
Boronic acid scope in the Pd-catalyzed cross-coupling of Pfp esters. Conditions: Ester (1.0 equiv), ArB(OH)_2_ (3.0 equiv), Na_2_CO_3_ (4.5 equiv), Pd_2_(dba)_3_ (3 mol%), PCy_3_HBF_4_ (12 mol%), dioxane (0.25 M), 120 °C, 15 h. See SI for details.

With optimized conditions in hand, the scope of the cross-coupling with respect to the boronic acid component was examined (Figure 1). We were pleased to find the reaction readily accommodates a range of electronically-diverse boronic acids, including neutral (**3a**) electron-rich (**3b, 3c**), electron-deficient bearing electrophilic carbonyl (**3d**), sterically-hindered (**3e**), as well as fluorinated boronic acids relevant from the medicinal chemistry standpoint (**3f**–**3i**). 

**Figure 2:**
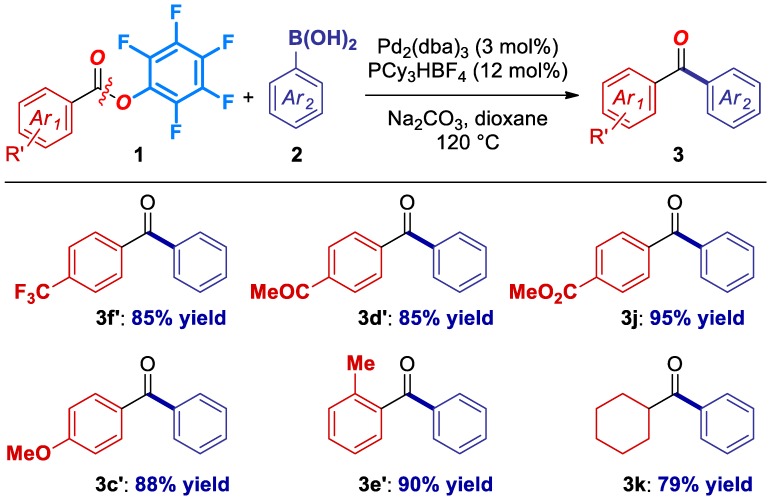
Ester scope in the Pd-catalyzed cross-coupling of Pfp esters. Conditions: Ester (1.0 equiv), ArB(OH)_2_ (3.0 equiv), Na_2_CO_3_ (4.5 equiv), Pd_2_(dba)_3_ (3 mol%), PCy3HBF4 (12 mol%), dioxane (0.25 M), 120 °C, 15 h. See SI for details.

We next explored the generality of this cross-coupling with respected to the ester electrophile (Figure 2). Pleasingly, the reaction tolerates electron-deficient substituents (**3f’**, **3d’**, **3j**), including electrophilic carbonyls (**3d’**, **3j**), electron-rich deactivating substituents (**3c’**), sterically-hindered (**3e’**), as well as aliphatic pfp ester precursors (**3k**). It is worthwhile to note that the reaction proceeded with full selectivity for the cross-coupling of a pfp ester in the presence of an aliphatic ester (**3j**), as expected from the C–O isomerization and our design (*vide infra*) [15,19].

**Scheme 2:**
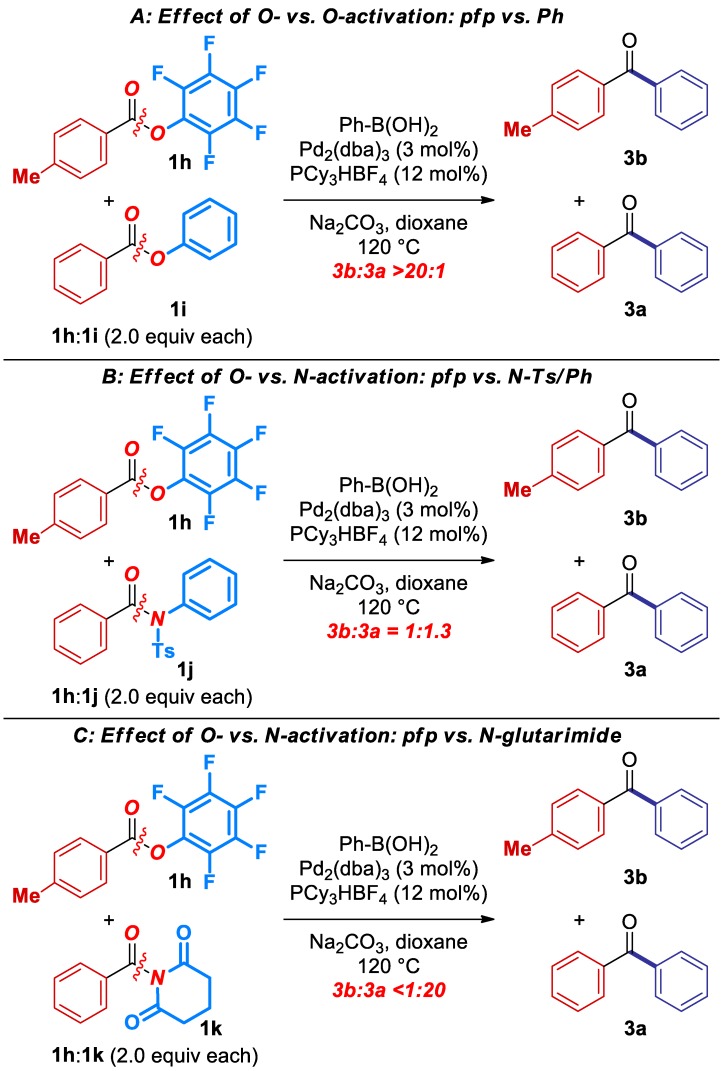
Competition experiments.

Next, to emphasize the synthetic utility of this transformation, we conducted a series of competition experiments between pfp esters and ester and amide electrophiles previously established in cross-coupling protocols (Scheme 2) [3,4,5,6,7,8]. *Most importantly, as expected on the basis of C–O isomerization, the reaction is fully selective for the cross-coupling in the presence of an activated phenolic ester (*Scheme 2A). A separate experiment using phenyl benzoate under the optimized conditions for the cross-coupling of pfp esters resulted in a quantitative recovery of PhCO_2_Ph.

**Scheme 3:**
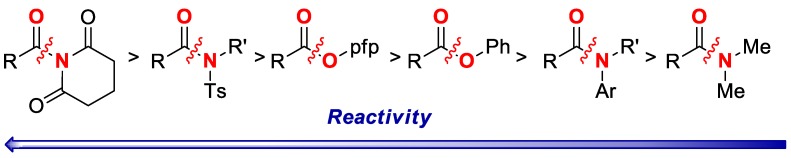
Reactivity scale in C(acyl)–N and C(acyl)–O Suzuki-Miyaura cross-coupling. Note that thus far only *N*-Acyl-glutarimides, *N*-Ts-sulfonamides and O-pfp esters have been shown to react with Pd-PR_3_ catalytic systems [1,2,3]. The reactivity of OPh esters, N-Ar amides and N-Me amides is based on Pd-NHC catalysts [1,15].

Furthermore, the reaction is slightly less selective for the cross-coupling of pfp esters cf. N-Ts sulfonamides (Scheme 2B; Ts/Ph:pfp = 1.3:1), whereas full selectivity is observed in the cross-coupling of *N*-acylglutarimides vs. pfp esters (Scheme 2C; >20:1), as expected on the basis of amide bond destabilization [6,11]. Overall, the competition experiments demonstrate high chemoselectivity of the cross-coupling of pfp esters, and permit to establish a unified reactivity scale in cross-coupling of esters and amides catalyzed by Pd-phosphanes (Scheme 3). It is well-established that cross-coupling of anilides is performed in the presence of *N*,*N*-dialkylamides [12].

To gain insight into the reaction mechanism additional experiments were conducted (not shown). (1) Competition experiments with differently substituted pfp esters revealed that electron-deficient arenes are more reactive (4-CF_3_:4-MeO > 20:1); while (2) differently substituted boronic acids revealed a small preference for electron-rich boronic acids (4-MeO:4-CF_3_ = 1.1:1). Overall, these findings suggest that Pd insertion may be the rate limiting step in this reaction.

**Scheme 4:**
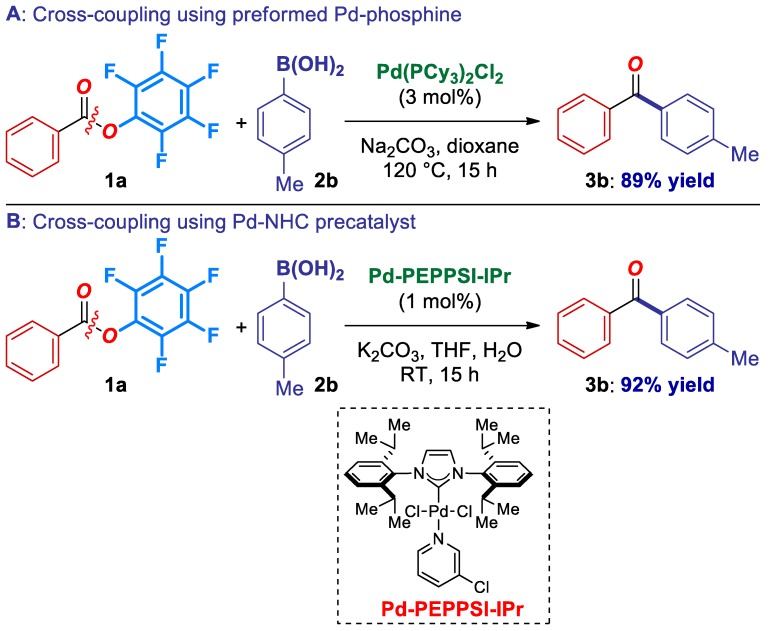
Cross-coupling using preformed Pd-phosphine and Pd(II)-NHC precatalysts.

Finally, we demonstrated that catalytic systems in the Suzuki-Miyaura cross-coupling of pfp esters are not limited to the in situ formed Pd(0)-phosphane catalysts. For example, preformed Pd-phosphane catalysts [25], as well as Pd(II)-NHCs [1], such as Pd(PCy_3_)_2_Cl_2_ and Pd-PEPPSI-IPr afford the coupling product in excellent yields (Scheme 4), highlighting the generality and rich synthetic potential of pentafluorophenyl esters as electrophiles in transition-metal catalysis. Future work will focus on the expansion of the catalyst portfolio in the cross-coupling of activated esters. With the availability of various catalyst systems, the pfp reagents should expand the implementation of ester C–O cross-coupling in organic synthesis [14,15,16,17,18].

## 3. Discussion

In summary, we have reported the Suzuki-Miyaura cross-coupling of pentafluorophenyl esters. The reaction is notable for the first use of Pd-phosphane catalysis in chemoselective Suzuki-Miyaura ester coupling by C–O cleavage. Furthermore, this method introduces pfp esters as new, ester-based, electrophilic reagents for transition-metal catalyzed cross-coupling reactions. Given the broad utility of pfp esters in nucleophilic addition reactions, we believe that these reagents will find wide application in the cross-coupling chemistry. In particular, this study highlights the utility of ground-state destabilization of acyl electrophiles to achieve chemoselective bond activation. Since pentafluorophenyl esters are easily prepared, bench-stable solids, and highly reactive, these reagents should be considered along phenolic esters in the future development of cross-coupling reactions by acyl [1,2,3] and decarbonylative pathways [2,7,23,24]. 

## 4. Materials and Methods

### 4.1. General Information

General methods have been published [13].

### 4.2. General Procedure for Cross-Coupling of Pentafluorophenyl Esters

An oven-dried vial equipped with a stir bar was charged with an ester substrate (neat, 1.0 equiv), boronic acid (typically, 3.0 equiv), sodium carbonate (typically, 4.5 equiv), Pd_2_(dba)_3_ (typically, 3 mol%), and PCy_3_HBF_4_ (typically, 12 mol%), placed under a positive pressure of argon, and subjected to three evacuation/backfilling cycles under high vacuum. Dioxane (typically, 0.25 M) was added with vigorous stirring at room temperature, the reaction mixture was placed in a preheated oil bath at 120 °C, and stirred for the indicated time at 120 °C. After the indicated time, the reaction mixture was cooled down to room temperature, diluted with CH_2_Cl_2_ (10 mL), filtered, and concentrated. The sample was analyzed by ^1^H-NMR (CDCl_3_, 500 MHz) and GC-MS to obtain conversion, selectivity and yield using internal standard and comparison with authentic samples. Purification by chromatography afforded the pure product.

### 4.3. Representative Procedure for Cross-Coupling of Pentafluorophenyl Esters

An oven-dried vial equipped with a stir bar was charged with perfluorophenyl benzoate (neat, 288.2 mg, 1.0 mmol), *p*-tolylboronic acid (408.0 mg, 3.0 mmol, 3.0 equiv), Na_2_CO_3_ (477.0 mg, 4.5 mmol, 4.5 equiv), Pd_2_(dba)_3_ (27.5 mg, 0.03 mmol, 3 mol%), and PCy_3_HBF_4_ (44.2 mg, 0.12 mmol, 12 mol%) placed under a positive pressure of argon, and subjected to three evacuation/backfilling cycles under high vacuum. Dioxane (0.25 M) was added with vigorous stirring at room temperature, the reaction mixture was placed in a preheated oil bath at 120 °C, and stirred for 15 h at 120 °C. After the indicated time, the reaction mixture was cooled down to room temperature, diluted with CH_2_Cl_2_ (10 mL), filtered, and concentrated. A sample was analyzed by ^1^H-NMR (CDCl_3_, 500 MHz) and GC-MS to obtain conversion, yield and selectivity using internal standard and comparison with authentic samples. Purification by chromatography on silica gel (hexanes/ethyl acetate) afforded the title product. Yield 86% (168.5 mg). White solid. Characterization data are included in the section below.

### 4.4. Characterization Data for Products **3a**–**3k** (Figures 1 and 2)

*Benzophenone* (**3a**). White solid. ^1^H-NMR (500 MHz, CDCl_3_) δ 7.83 (d, *J* = 8.9 Hz, 4 H), 7.62 (t, *J* = 7.4 Hz, 2 H), 7.51 (t, *J* = 7.6 Hz, 4 H). ^13^C-NMR (125 MHz, CDCl_3_) δ 196.75, 137.61, 132.42, 130.07, 128.28.

*Phenyl(p-tolyl)methanone* (**3b**). White solid. ^1^H-NMR (500 MHz, CDCl_3_) δ 7.81 (d, *J* = 7.7 Hz, 2 H), 7.75 (d, *J* = 7.5 Hz, 2 H), 7.60 (t, *J* = 7.4 Hz, 1 H), 7.50 (t, *J* = 7.2 Hz, 2 H), 7.31 (d, *J* = 7.7 Hz, 2 H), 2.47 (s, 3 H). ^13^C-NMR (125 MHz, CDCl_3_) δ 196.49, 143.22, 137.98, 134.90, 132.14, 130.31, 129.93, 128.97, 128.20, 21.66.

*(4-Methoxyphenyl)(phenyl)methanone* (**3c**). White solid. ^1^H-NMR (500 MHz, CDCl_3_) δ 7.86 (d, *J* = 8.0 Hz, 2 H), 7.78 (d, *J* = 7.6 Hz, 2 H), 7.59 (t, *J* = 7.3 Hz, 1 H), 7.50 (t, *J* = 7.4 Hz, 2 H), 6.99 (d, *J* = 8.0 Hz, 2 H), 3.92 (s, 3 H). ^13^C-NMR (125 MHz, CDCl_3_) δ 195.56, 163.23, 138.30, 132.57, 131.89, 130.17, 129.74, 128.19, 113.56, 55.51.

*1-(4-Benzoylphenyl)ethan-1-one* (**3d**). White solid. ^1^H-NMR (500 MHz, CDCl_3_) δ 8.09 (d, *J* = 8.2 Hz, 2 H), 7.89 (d, *J* = 8.2 Hz, 2 H), 7.83 (d, *J* = 7.5 Hz, 2 H), 7.65 (t, *J* = 7.4 Hz, 1 H), 7.53 (t, *J* = 7.7 Hz, 2 H), 2.70 (s, 3 H). ^13^C-NMR (125 MHz, CDCl_3_) δ 197.52, 195.96, 139.57, 136.92, 133.00, 130.11, 130.05, 128.49, 128.17, 26.92.

*Phenyl(o-tolyl)methanone* (**3e**). White solid. ^1^H-NMR (500 MHz, CDCl_3_) δ 7.83 (d, *J* = 7.7 Hz, 2 H), 7.60 (d, *J* = 6.9 Hz, 1 H), 7.49 (t, *J* = 7.6 Hz, 2 H), 7.42 (t, *J* = 7.5 Hz, 1 H), 7.37 –7.30 (m, 2 H), 7.30–7.27 (m, 1 H), 2.36 (s, 3 H). ^13^C-NMR (125 MHz, CDCl_3_) δ 198.64, 138.63, 137.75, 136.75, 133.14, 131.00, 130.24, 130.14, 128.52, 128.46, 125.20, 20.00.

*Phenyl(4-(trifluoromethyl)phenyl)methanone* (**3f**). White solid. ^1^H-NMR (500 MHz, CDCl_3_) δ 7.93 (d, *J* = 8.0 Hz, 2 H), 7.84 (d, *J* = 7.7 Hz, 2 H), 7.79 (d, *J* = 8.0 Hz, 2 H), 7.66 (t, *J* = 7.4 Hz, 1 H), 7.54 (t, *J* = 7.6 Hz, 2 H). ^13^C-NMR (125 MHz, CDCl_3_) δ 195.53, 140.74, 136.74, 133.73 (*J^F^* = 32.5 Hz), 133.09, 130.14, 130.11, 128.54, 125.36 (*J^F^* = 7.5 Hz), 123.70 (*J^F^* = 273.0 Hz). ^19^F-NMR (471 MHz, CDCl_3_) δ 63.41.

*Phenyl(3-(trifluoromethyl)phenyl)methanone* (**3g**). White solid. ^1^H-NMR (500 MHz, CDCl_3_) δ 8.07 (s, 1 H), 7.98 (d, *J* = 7.8 Hz, 1 H), 7.85 (d, *J* = 8.0 Hz, 1 H), 7.80 (d, *J* = 7.7 Hz, 2 H), 7.63 (t, *J* = 7.6 Hz, 2 H), 7.52 (t, *J* = 7.6 Hz, 2 H). ^13^C-NMR (125 MHz, CDCl_3_) δ 195.32, 138.45, 136.92, 133.25, 133.14, 131.17 (*J^F^* = 32.7 Hz), 130.16, 129.09, 128.97 (*J^F^* = 7.5 Hz), 128.71, 126.84 (*J^F^* = 8.8 Hz), 123.84 (*J^F^* = 272.9 Hz). ^19^F-NMR (471 MHz, CDCl_3_) δ 62.77.

*(4-Fluorophenyl)(phenyl)methanone* (**3h**). White solid. ^1^H-NMR (500 MHz, CDCl_3_) δ 7.90–7.84 (m, 2 H), 7.79 (d, *J* = 7.7 Hz, 2 H), 7.62 (t, *J* = 6.9 Hz, 1 H), 7.51 (t, *J* = 7.4 Hz, 2 H), 7.18 (t, *J* = 8.2 Hz, 2 H). ^13^C-NMR (125 MHz, CDCl_3_) δ 195.26, 165.39 (*J^F^* = 254.1 Hz), 137.51, 133.81 (*J^F^* = 2.5 Hz), 132.67 (*J^F^* = 8.8 Hz), 132.47, 129.88, 128.36, 115.45 (*J^F^* = 21.4 Hz). ^19^F-NMR (471 MHz, CDCl_3_) δ 105.98.

*(3,5-Difluorophenyl)(phenyl)methanone* (**3i**). White solid. ^1^H-NMR (500 MHz, CDCl_3_) δ 7.35 (dt, *J* = 40.7, 18.4 Hz, 4 H), 7.03–6.87 (m, 1 H), 6.40 (d, *J* = 41.0 Hz, 2 H), 6.13 (d, *J* = 40.9 Hz, 2 H). ^13^C-NMR (125 MHz, CDCl_3_) δ 193.95, 162.74 (*J^F^* = 250.3 Hz), 162.65 (*J^F^* = 251.6 Hz), 136.40, 133.16, 129.98, 128.59, 112.96 (*J^F^* = 20.1 Hz), 107.73 (*J^F^* = 25.8 Hz). ^19^F-NMR (471 MHz, CDCl_3_) δ 108.15.

*Methyl 4-benzoylbenzoate* (**3j**). White solid. ^1^H-NMR (500 MHz, CDCl_3_) δ 8.17 (d, *J* = 8.2 Hz, 2 H), 7.87 (d, *J* = 8.2 Hz, 2 H), 7.83 (d, *J* = 7.5 Hz, 2 H), 7.64 (t, *J* = 7.4 Hz, 1 H), 7.53 (t, *J* = 7.6 Hz, 2 H), 3.99 (s, 3 H). ^13^C-NMR (125 MHz, CDCl_3_) δ 196.03, 166.32, 141.33, 136.96, 133.22, 132.95, 130.11, 129.78, 129.50, 128.47, 52.48.

*Cyclohexyl(phenyl)methanone* (**3k**). White solid. ^1^H-NMR (500 MHz, CDCl_3_) δ 7.98–7.96 (d, *J* = 8.2 Hz, 2 H), 7.58–7.56 (t, *J* = 7.5 Hz, 1 H), 7.50–7.47 (t, *J* = 7.7 Hz, 2 H), 3.31–3.27 (t, *J* = 11.5 Hz, 1 H), 1.93–1.86 (m, 4 H), 1.78–1.75 (d, *J* = 11.7 Hz, 1 H), 1.54–1.49 (t, *J* = 13.4 Hz, 2 H), 1.46–1.39 (m, 2 H), 1.34–1.31 (d, *J* = 12.5 Hz, 1 H). ^13^C-NMR (125 MHz, CDCl_3_) δ 203.92, 136.38, 132.73, 128.59, 128.27, 45.65, 29.44, 25.98, 25.88.

## Figures and Tables

**Table 1:**
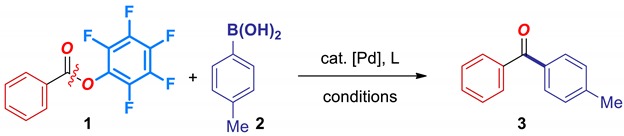
Optimization of the Suzuki-Miyaura cross-coupling of Pfp esters. ^1^

Entry	Catalyst	Ligand	Base	[Pd]:L	Yield (%)
1	Pd(OAc)_2_	PCy_3_HBF_4_	Na_2_CO_3_	1:4	52
2	Pd(OAc)_2_	PCy_3_HBF_4_	KHCO_3_	1:4	53
3	Pd(OAc)_2_	PCy_3_HBF_4_	NaHCO_3_	1:4	30
4	Pd(OAc)_2_	PCy_3_HBF_4_	K_2_CO_3_	1:4	12
5	Pd(OAc)_2_	PCy_3_HBF_4_	K_3_PO_4_	1:4	47
6	Pd(OAc)_2_	PCy_3_HBF_4_	KF	1:4	56
7	Pd(OAc)_2_	PPhCy_2_	Na_2_CO_3_	1:4	60
8	Pd(OAc)_2_	PPh_2_Cy	Na_2_CO_3_	1:4	5
9	Pd(OAc)_2_	PPh_3_	Na_2_CO_3_	1:4	5
10	Pd(OAc)_2_	DPPB	Na_2_CO_3_	1:4	13
11	Pd(OAc)_2_	Xantphos	Na_2_CO_3_	1:4	<5
12	Pd(OAc)_2_	Pt-Bu_3_HBF_4_	Na_2_CO_3_	1:4	<5
13	Pd(dba)_2_	PCy_3_HBF_4_	Na_2_CO_3_	1:4	23
14	PdCl_2_	PCy_3_HBF_4_	Na_2_CO_3_	1:4	25
15	Pd_2_(dba)_3_	PCy_3_HBF_4_	Na_2_CO_3_	1:2	74
16 ^2^	Pd_2_(dba)_3_	PCy_3_HBF_4_	Na_2_CO_3_	1:2	85
17 ^3^	Pd_2_(dba)_3_	PCy_3_HBF_4_	Na_2_CO_3_	1:1	44
18 ^4^	Pd_2_(dba)_3_	PCy_3_HBF_4_	Na_2_CO_3_	2:1	30
19 ^5^	Pd(OAc)_2_	PCy_3_HBF_4_	Na_2_CO_3_	1:4	83
20 ^5^	Pd_2_(dba)_3_	PCy_3_HBF_4_	Na_2_CO_3_	1:2	92
21 ^6^	Pd_2_(dba)_3_	PCy_3_HBF_4_	Na_2_CO_3_	1:2	75
22 ^7^	Pd_2_(dba)_3_	PCy_3_HBF_4_	Na_2_CO_3_	1:2	89

^1^ Conditions: Ester (1.0 equiv), 4-Tol-B(OH)_2_ (2.0 equiv), base (2.5 equiv), [Pd] (3 mol%), ligand (12 mol%), solvent (0.25 M), 120 °C, 15 h. ^2^ [Pd] (1.5 mol%), ligand (12 mol%), 4-Tol-B(OH)_2_ (3.0 equiv), base (4.5 equiv). ^3^ [Pd] (3 mol%), ligand (6 mol%), 4-Tol-B(OH)_2_ (3.0 equiv), base (4.5 equiv). ^4^ [Pd] (3 mol%), ligand (3 mol%), 4-Tol-B(OH)_2_ (3.0 equiv), base (4.5 equiv). ^5^ 4-Tol-B(OH)_2_ (3.0 equiv), base (4.5 equiv). ^6^ Dioxane (0.10 M). ^7^ Dioxane (0.50 M).

## Supplementary Materials

Experimental procedures and characterization data are available online.
