# Supplementary material for: Pd-Catalyzed Suzuki-Miyaura Cross-Coupling of Pentafluorophenyl Esters
Source: Molecules. 2018 Nov 29;23(12):3134. doi: 10.3390/molecules23123134 (PMC6321476; doi:10.3390/molecules23123134)

Pd-Catalyzed Suzuki–Miyaura Cross-Coupling of Pentafluorophenyl Esters

Jonathan Buchspies,[†] Daniel J. Pyle,[†] Huixin He,[†,*] and Michal Szostak[†,*]

[†]*Department of Chemistry, Rutgers University, 73 Warren Street, Newark, New Jersey 07102, United States*

michal.szostak@rutgers.edu; *huixinhe@rutgers.edu*

Supporting Information

Table of Contents 1

List of Known Compounds/General Methods 2
Experimental Procedures and Characterization Data 3
References 7
^{1}H and ^{13}C NMR Spectra 8

Corresponding Author:
Prof. Dr. M. Szostak, Prof. Dr. Huixin He
Department of Chemistry, Rutgers University
73 Warren Street, Newark, NJ 07102, United States
E-mail: michal.szostak@rutgers.edu

List of Known Compounds/General Methods

All starting materials reported in the manuscript have been prepared according to the method reported previously.[1-3] All experiments involving palladium were performed using standard Schlenk techniques under argon or nitrogen atmosphere unless stated otherwise. All solvents were purchased at the highest commercial grade and used as received or after purification by passing through activated alumina columns or distillation from sodium/benzophenone under nitrogen. All solvents were deoxygenated prior to use. All other chemicals were purchased at the highest commercial grade and used as received. Reaction glassware was oven-dried at 140 °C for at least 24 h or flame-dried prior to use, allowed to cool under vacuum and purged with argon (three cycles). All products were identified using ^{1}H NMR analysis and comparison with authentic samples. GC and/or GC/MS analysis was used for volatile products. ^{1}H NMR and ^{13}C NMR spectra were recorded in $CDCl_3$ on Bruker spectrometers at 500 (^{1}H NMR) and 125 MHz (^{13}C NMR). All shifts are reported in parts per million (ppm) relative to residual $CHCl_3$ peak (7.27 and 77.2 ppm, ^{1}H NMR and ^{13}C NMR, respectively). All coupling constants (*J*) are reported in hertz (Hz). Abbreviations are: s, singlet; d, doublet; t, triplet; q, quartet; brs, broad singlet. GC-MS chromatography was performed using Agilent HP6890 GC System and Agilent 5973A inert XL EI/CI MSD using helium as the carrier gas at a flow rate of 1 mL/min and an initial oven temperature of 50 °C. The injector temperature was 250 °C. The detector temperature was 250 °C. For runs with the initial oven temperature of 50 °C, temperature was increased with a 10 °C/min ramp after 50 °C hold for 3 min to a final temperature of 220 °C, then hold at 220 °C for 15 min (splitless mode of injection, total run time of 22.0 min). High-resolution mass spectra (HRMS) were measured on a 7T Bruker Daltonics FT-MS instrument (for HRMS). Melting point was measured on MeltEMP (laboratory devices). All flash chromatography was performed using silica gel, 60 Å, 300 mesh. TLC analysis was carried out on glass plates coated with silica gel 60 F254, 0.2 mm thickness. The plates were visualized using a 254 nm ultraviolet lamp or aqueous potassium permanganate solutions. ^{1}H NMR and ^{13}C NMR data are given for all compounds in the Supplementary Information. ^{1}H NMR, ^{13}C NMR and HRMS data are reported for all new compounds. All compounds reported in this manuscript have been previously reported, unless stated otherwise. Spectroscopic data matched literature values.

Experimental Procedures and Characterization Data

General Procedure for Cross-Coupling of Pentafluorophenyl Esters. An oven-dried vial equipped with a stir bar was charged with an ester substrate (neat, 1.0 equiv), boronic acid (typically, 3.0 equiv), sodium carbonate (typically, 4.5 equiv), $Pd_2(dba)_3$ (typically, 3 mol%), and PCy_3HBF_4 (typically, 12 mol%), placed under a positive pressure of argon, and subjected to three evacuation/backfilling cycles under high vacuum. Dioxane (typically, 0.25 M) was added with vigorous stirring at room temperature, the reaction mixture was placed in a preheated oil bath at 120 °C, and stirred for the indicated time at 120 °C. After the indicated time, the reaction mixture was cooled down to room temperature, diluted with CH_2Cl_2 (10 mL), filtered, and concentrated. The sample was analyzed by ^1H NMR ($CDCl_3$, 500 MHz) and GC-MS to obtain conversion, selectivity and yield using internal standard and comparison with authentic samples. Purification by chromatography afforded the pure product.

Representative Procedure for Cross-Coupling of Pentafluorophenyl Esters. An oven-dried vial equipped with a stir bar was charged with perfluorophenyl benzoate (neat, 288.2 mg, 1.0 mmol), *p*-tolylboronic acid (408.0 mg, 3.0 mmol, 3.0 equiv), Na_2CO_3 (477.0 mg, 4.5 mmol, 4.5 equiv), $Pd_2(dba)_3$ (27.5 mg, 0.03 mmol, 3 mol%), and PCy_3HBF_4 (44.2 mg, 0.12 mmol, 12 mol%) placed under a positive pressure of argon, and subjected to three evacuation/backfilling cycles under high vacuum. Dioxane (0.25 M) was added with vigorous stirring at room temperature, the reaction mixture was placed in a preheated oil bath at 120 °C, and stirred for 15 h at 120 °C. After the indicated time, the reaction mixture was cooled down to room temperature, diluted with CH_2Cl_2 (10 mL), filtered, and concentrated. A sample was analyzed by ^1H NMR ($CDCl_3$, 500 MHz) and GC-MS to obtain conversion, yield and selectivity using internal standard and comparison with authentic samples. Purification by chromatography on silica gel (hexanes/ethyl acetate) afforded the title product. Yield 86% (168.5 mg). White solid. Characterization data are included in the section below.

Characterization Data

*Characterization Data for Products **3a-3k** (Tables 2-3).*

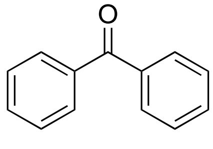

Benzophenone (3a). White solid. **^{1}H NMR (500 MHz, $CDCl_3$)** δ 7.83 (d, *J* = 8.9 Hz, 4 H), 7.62 (t, *J* = 7.4 Hz, 2 H), 7.51 (t, *J* = 7.6 Hz, 4 H). **^{13}C NMR (125 MHz, $CDCl_3$)** δ 196.75, 137.61, 132.42, 130.07, 128.28.

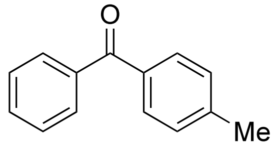

Phenyl(*p*-tolyl)methanone (3b). White solid. **^{1}H NMR (500 MHz, $CDCl_3$)** δ 7.81 (d, *J* = 7.7 Hz, 2 H), 7.75 (d, *J* = 7.5 Hz, 2 H), 7.60 (t, *J* = 7.4 Hz, 1 H), 7.50 (t, *J* = 7.2 Hz, 2 H), 7.31 (d, *J* = 7.7 Hz, 2 H), 2.47 (s, 3 H). **^{13}C NMR (125 MHz, $CDCl_3$)** δ 196.49, 143.22, 137.98, 134.90, 132.14, 130.31, 129.93, 128.97, 128.20, 21.66.

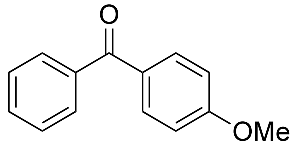

(4-Methoxyphenyl)(phenyl)methanone (3c). White solid. **^{1}H NMR (500 MHz, $CDCl_3$)** δ 7.86 (d, *J* = 8.0 Hz, 2 H), 7.78 (d, *J* = 7.6 Hz, 2 H), 7.59 (t, *J* = 7.3 Hz, 1 H), 7.50 (t, *J* = 7.4 Hz, 2 H), 6.99 (d, *J* = 8.0 Hz, 2 H), 3.92 (s, 3 H). **^{13}C NMR (125 MHz, $CDCl_3$)** δ 195.56, 163.23, 138.30, 132.57, 131.89, 130.17, 129.74, 128.19, 113.56, 55.51.

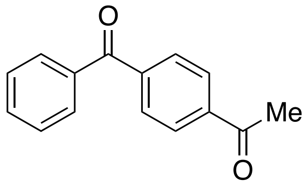

1-(4-Benzoylphenyl)ethan-1-one (3d). White solid. **^{1}H NMR (500 MHz, $CDCl_3$)** δ 8.09 (d, *J* = 8.2 Hz, 2 H), 7.89 (d, *J* = 8.2 Hz, 2 H), 7.83 (d, *J* = 7.5 Hz, 2 H), 7.65 (t, *J* = 7.4 Hz, 1 H), 7.53 (t, *J* = 7.7 Hz, 2 H), 2.70 (s, 3 H). **^{13}C NMR (125 MHz, $CDCl_3$)** δ 197.52, 195.96, 139.57, 136.92, 133.00, 130.11, 130.05, 128.49, 128.17, 26.92.

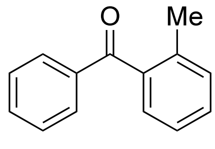

Phenyl(*o*-tolyl)methanone (3e). White solid. **^{1}H NMR (500 MHz, $CDCl_3$)** δ 7.83 (d, *J* = 7.7 Hz, 2 H), 7.60 (d, *J* = 6.9 Hz, 1 H), 7.49 (t, *J* = 7.6 Hz, 2 H), 7.42 (t, *J* = 7.5 Hz, 1 H), 7.37-7.30 (m, 2 H), 7.30-7.27 (m, 1 H), 2.36 (s, 3 H). **^{13}C NMR (125 MHz, $CDCl_3$)** δ 198.64, 138.63, 137.75, 136.75, 133.14, 131.00, 130.24, 130.14, 128.52, 128.46, 125.20, 20.00.

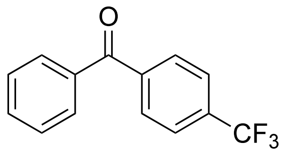

Phenyl(4-(trifluoromethyl)phenyl)methanone (3f). White solid. **^{1}H NMR (500 MHz, $CDCl_3$)** δ 7.93 (d, *J* = 8.0 Hz, 2 H), 7.84 (d, *J* = 7.7 Hz, 2 H), 7.79 (d, *J* = 8.0 Hz, 2 H), 7.66 (t, *J* = 7.4 Hz, 1 H), 7.54 (t, *J* = 7.6 Hz, 2 H). **^{13}C NMR (125 MHz, $CDCl_3$)** δ 195.53, 140.74, 136.74, 133.73 (J^F = 32.5 Hz), 133.09, 130.14, 130.11, 128.54, 125.36 (J^F = 7.5 Hz), 123.70 (J^F = 273.0 Hz). **^{19}F NMR (471 MHz, $CDCl_3$)** δ -63.41.

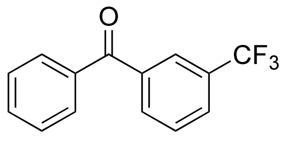

Phenyl(3-(trifluoromethyl)phenyl)methanone (3g). White solid. **^{1}H NMR (500 MHz, $CDCl_3$)** δ 8.07 (s, 1 H), 7.98 (d, *J* = 7.8 Hz, 1 H), 7.85 (d, *J* = 8.0 Hz, 1 H), 7.80 (d, *J* = 7.7 Hz, 2 H), 7.63 (t, *J* = 7.6 Hz, 2 H), 7.52 (t, *J* = 7.6 Hz, 2 H). **^{13}C NMR (125 MHz, $CDCl_3$)** δ 195.32, 138.45, 136.92, 133.25, 133.14, 131.17 (J^F = 32.7 Hz), 130.16, 129.09, 128.97 (J^F = 7.5 Hz), 128.71, 126.84 (J^F = 8.8 Hz), 123.84 (J^F = 272.9 Hz). **^{19}F NMR (471 MHz, $CDCl_3$)** δ -62.77.

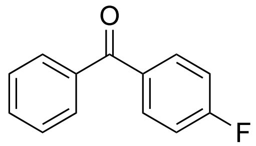

(4-Fluorophenyl)(phenyl)methanone (3h). White solid. **^{1}H NMR (500 MHz, $CDCl_3$)** δ 7.90-7.84 (m, 2 H), 7.79 (d, *J* = 7.7 Hz, 2 H), 7.62 (t, *J* = 6.9 Hz, 1 H), 7.51 (t, *J* = 7.4 Hz, 2 H), 7.18 (t, *J* = 8.2 Hz, 2 H). **^{13}C NMR (125 MHz, $CDCl_3$)** δ 195.26, 165.39 (J^F = 254.1 Hz), 137.51, 133.81 (J^F = 2.5 Hz), 132.67 (J^F = 8.8 Hz), 132.47, 129.88, 128.36, 115.45 (J^F = 21.4 Hz). **^{19}F NMR (471 MHz, $CDCl_3$)** δ -105.98.

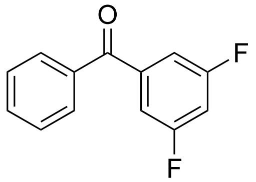

(3,5-Difluorophenyl)(phenyl)methanone (3i). White solid. **^{1}H NMR (500 MHz, $CDCl_3$)** δ 7.35 (dt, *J* = 40.7, 18.4 Hz, 4 H), 7.03-6.87 (m, 1 H), 6.40 (d, *J* = 41.0 Hz, 2 H), 6.13 (d, *J* = 40.9 Hz, 2 H). **^{13}C NMR (125 MHz, $CDCl_3$)** δ 193.95, 162.74 (J^F = 250.3 Hz), 162.65 (J^F = 251.6 Hz), 136.40, 133.16, 129.98, 128.59, 112.96 (J^F = 20.1 Hz), 107.73 (J^F = 25.8 Hz). **^{19}F NMR (471 MHz, $CDCl_3$)** δ -108.15.

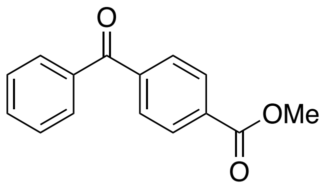

Methyl 4-benzoylbenzoate (3j). White solid. **^{1}H NMR (500 MHz, $CDCl_3$)** δ 8.17 (d, *J* = 8.2 Hz, 2 H), 7.87 (d, *J* = 8.2 Hz, 2 H), 7.83 (d, *J* = 7.5 Hz, 2 H), 7.64 (t, *J* = 7.4 Hz, 1 H), 7.53 (t, *J* = 7.6 Hz, 2 H), 3.99 (s, 3 H). **^{13}C NMR (125 MHz, $CDCl_3$)** δ 196.03, 166.32,

141.33, 136.96, 133.22, 132.95, 130.11, 129.78, 129.50, 128.47, 52.48.

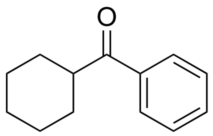

Cyclohexyl(phenyl)methanone (3k). White solid. **^{1}H NMR (500 MHz, $CDCl_3$)** δ 7.98-7.96 (d, *J* = 8.2 Hz, 2 H), 7.58-7.56 (t, *J* = 7.5 Hz, 1 H), 7.50-7.47 (t, *J* = 7.7 Hz, 2 H), 3.31-3.27 (t, *J* = 11.5 Hz, 1 H), 1.93-1.86 (m, 4 H), 1.78-1.75 (d, *J* = 11.7 Hz, 1 H), 1.54-1.49 (t, *J* = 13.4 Hz, 2 H), 1.46-1.39 (m, 2 H), 1.34-1.31 (d, *J* = 12.5 Hz, 1 H). **^{13}C NMR (125 MHz, $CDCl_3$)** δ 203.92, 136.38, 132.73, 128.59, 128.27, 45.65, 29.44, 25.98, 25.88.

References

1. (a) Lei, P.; Meng, G.; Shi, S.; Ling, Y.; An, J.; Szostak, R.; Szostak, M. *Chem. Sci.* **2017**, *8*, 6525. (b) Li, G.; Shi, S.; Szostak, M. *Adv. Synth. Catal.* **2018**, *360*, 1538.
2. (a) Halima, T. B.; Zhang, W.; Yalaoui, I.; Hong, X.; Yang, Y. F.; Houk, K. N.; Newman, S. G. *J. Am. Chem. Soc.* **2017**, *139*, 1311. (b) Muto, K.; Yamaguchi, J.; Musaev, D. G.; Itami, K. *Nat. Commun.* **2015**, *6*, 7508.
3. For additional examples, see: (a) Zhao, H.; Burke, Jr., T. R. *Tetrahedron* **1997**, *53*, 4219. (b) Adamczyk, M.; Fishpaugh, J. R.; Heuser, K. J. *Bioconjugate Chem.* **1997**, *8*, 253. (c) Atherton, E.; Cameron, L. R.; Sheppard, R. C. *Tetrahedron* **1988**, *44*, 843.

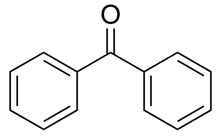
O

3a

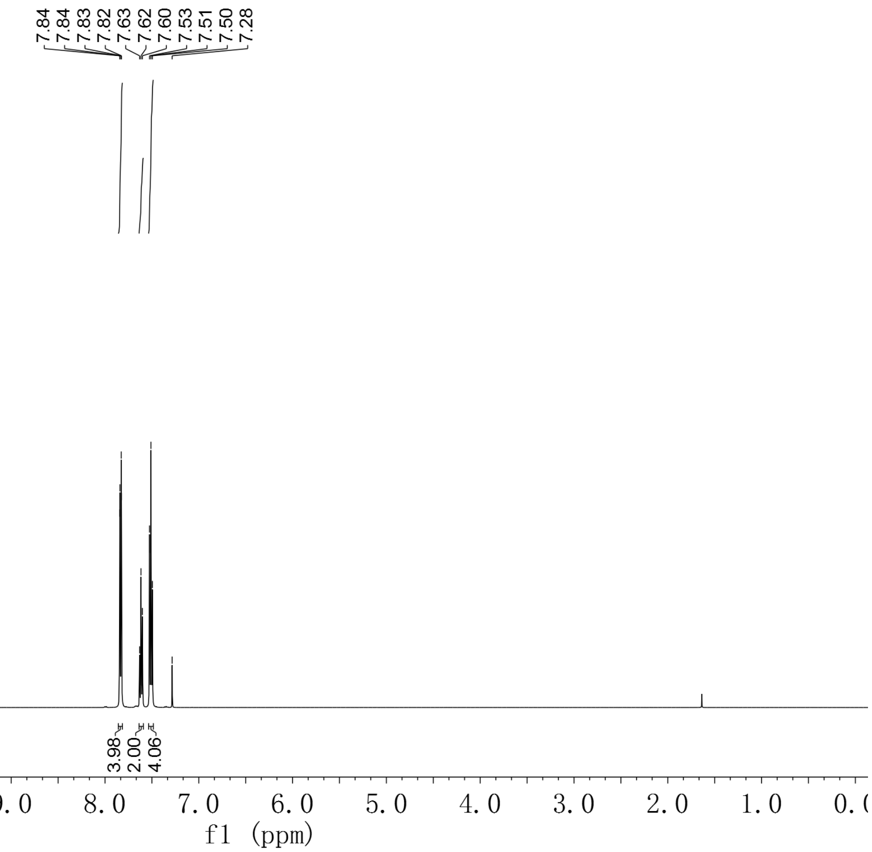
7.84
7.84
7.83
7.82
7.63
7.62
7.60
7.53
7.51
7.50
7.28
3.98
2.00
4.06
12.0 11.0 10.0 9.0 8.0 7.0 6.0 5.0 4.0 3.0 2.0 1.0
f1 (ppm)

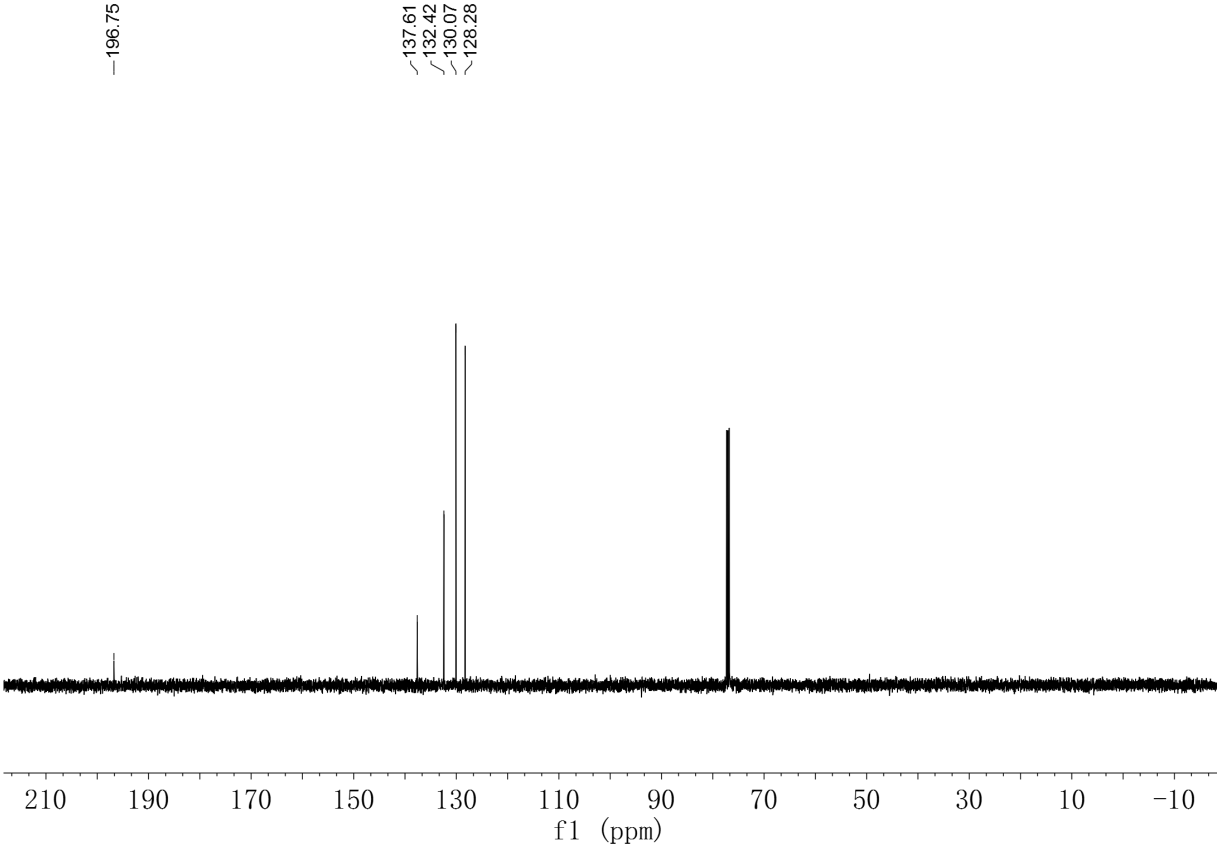
196.75
137.61
132.42
130.07
128.28
210 190 170 150 130 110 90 70 50 30 10 −10
f1 (ppm)

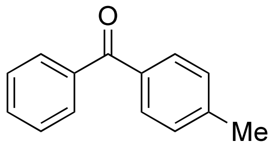
O
Me

3b

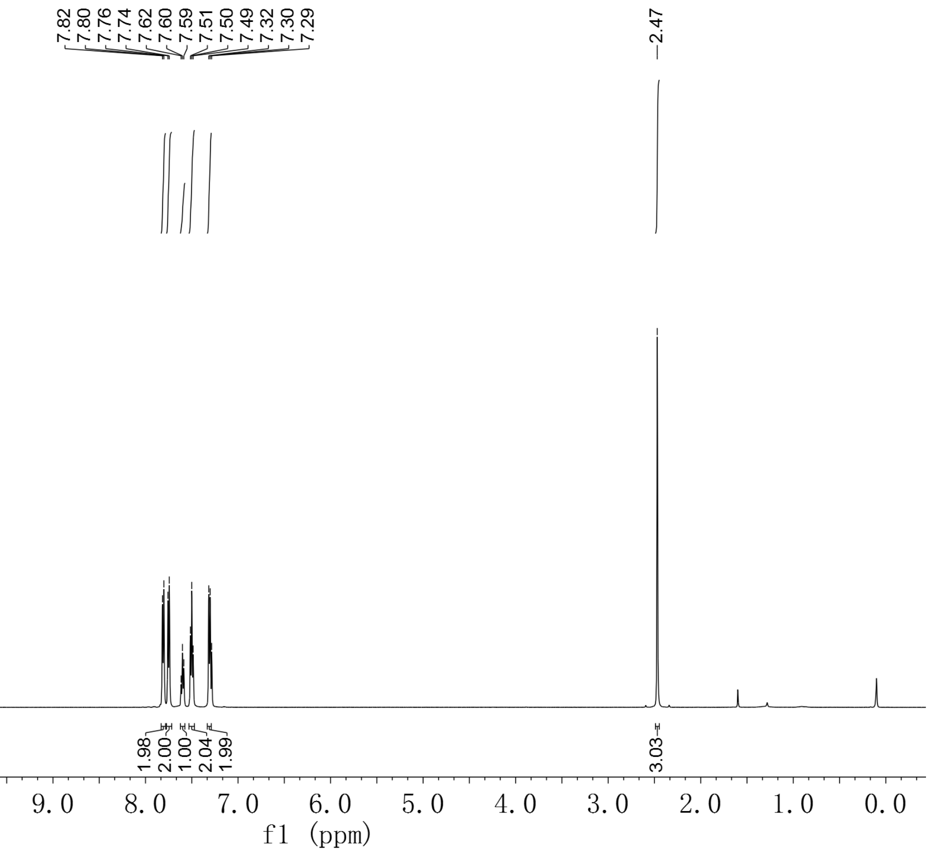
7.82
7.80
7.76
7.74
7.62
7.60
7.59
7.51
7.50
7.49
7.32
7.30
7.29
2.47
1.98
2.00
1.00
2.04
1.99
3.03
12.0 11.0 10.0 9.0 8.0 7.0 6.0 5.0 4.0 3.0 2.0 1.0 0.0
f1 (ppm)

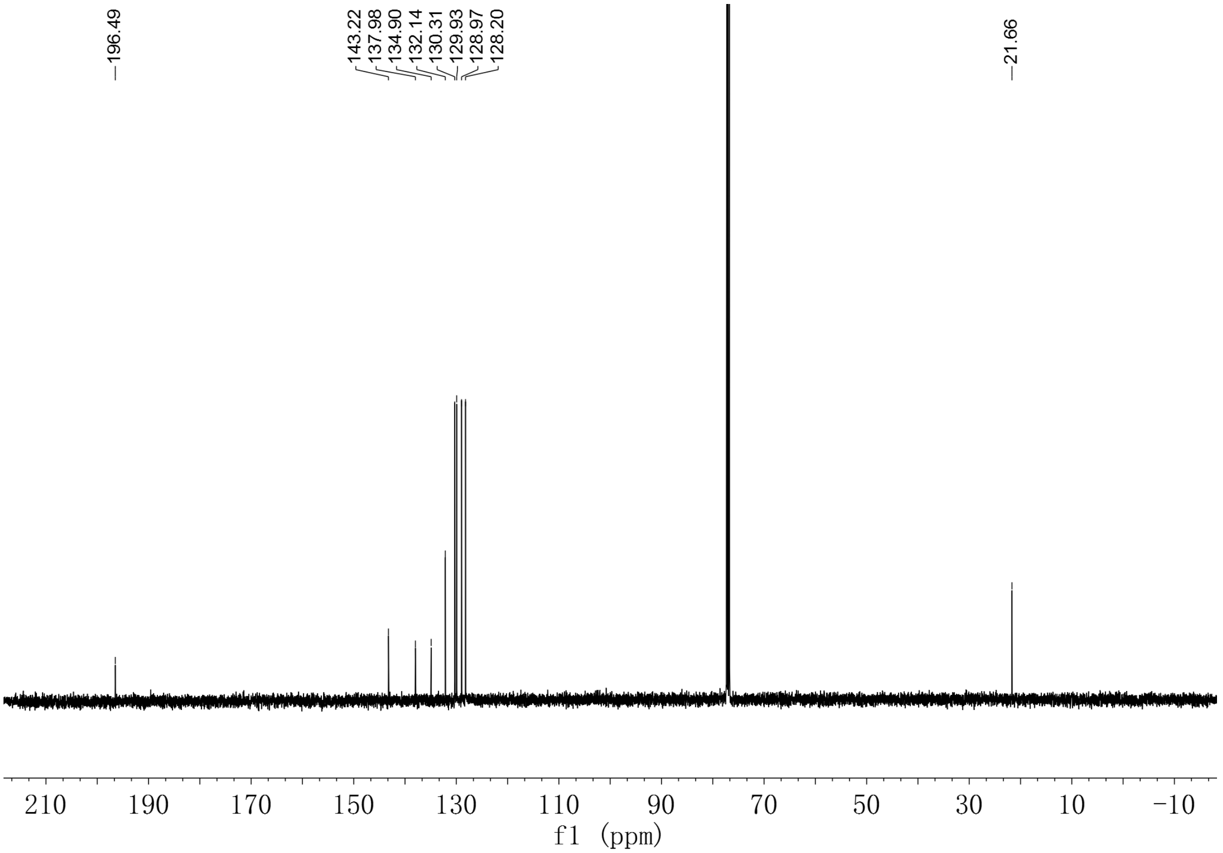
196.49
143.22
137.98
134.90
132.14
130.31
129.93
128.97
128.20
21.66
210 190 170 150 130 110 90 70 50 30 10 −10
f1 (ppm)

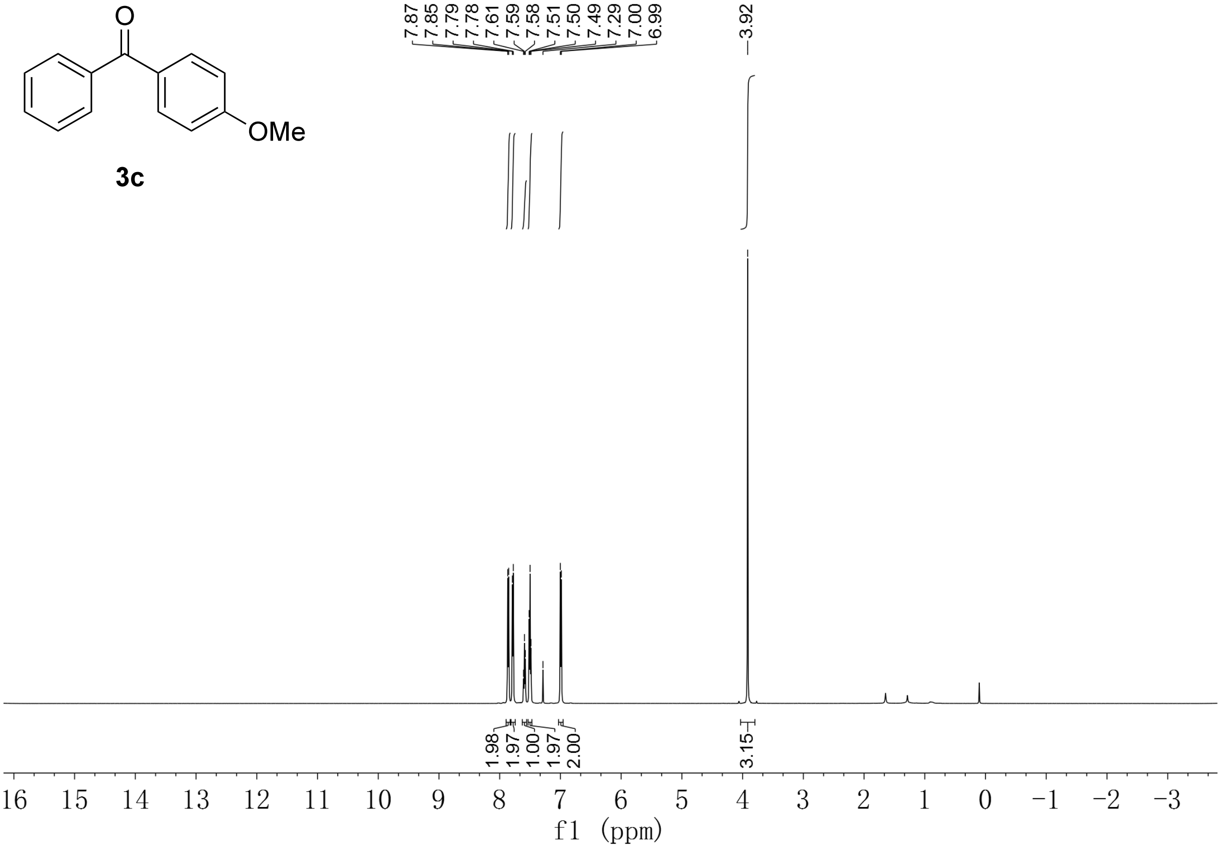
OMe
3c
7.87
7.85
7.79
7.78
7.61
7.59
7.58
7.51
7.50
7.49
7.29
7.00
6.99
3.92
1.98
1.97
1.00
1.97
2.00
3.15
16 15 14 13 12 11 10 9 8 7 6 5 4 3 2 1 0 −1 −2 −3
f1 (ppm)

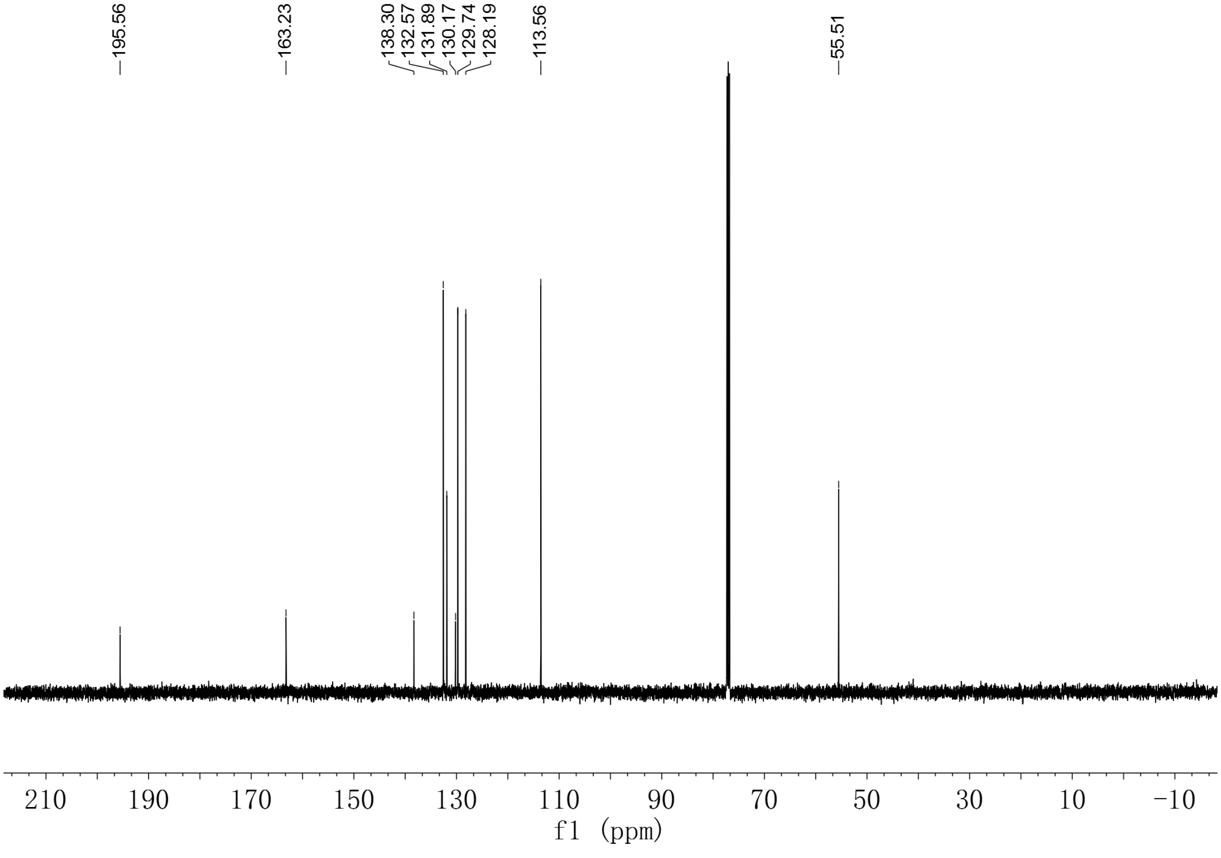
195.56
163.23
138.30
132.57
131.89
130.17
129.74
128.19
113.56
55.51
210 190 170 150 130 110 90 70 50 30 10 −10
f1 (ppm)

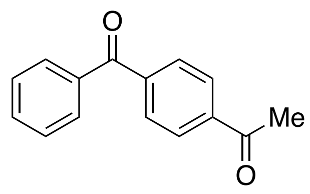
O
Me
O

3d

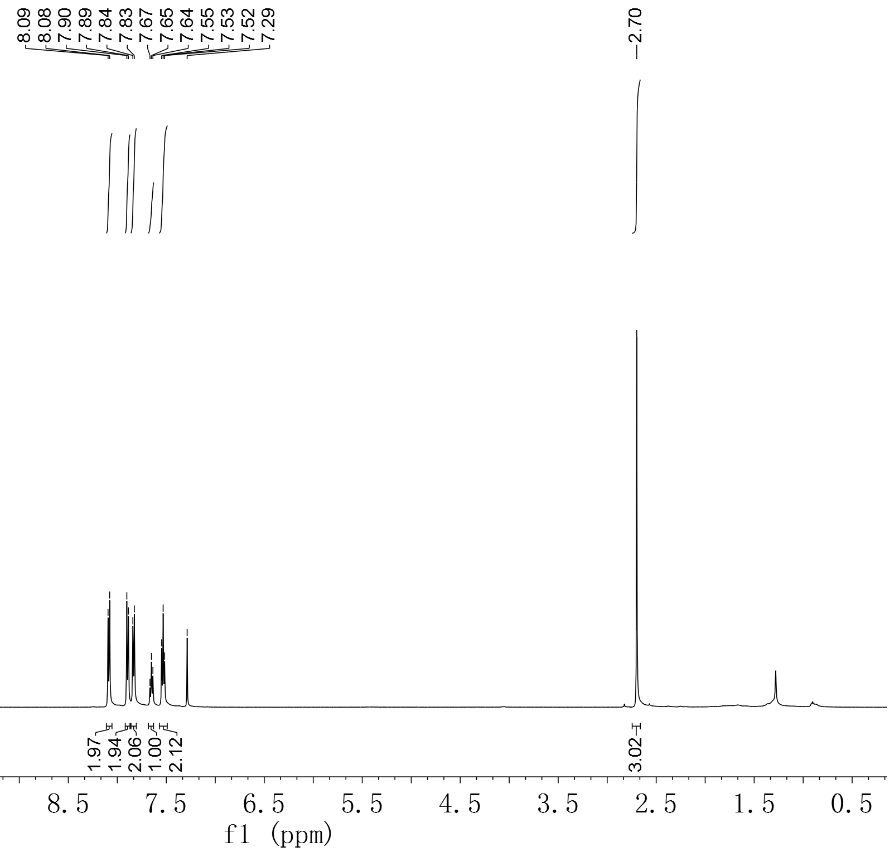
8.09
8.08
7.90
7.89
7.84
7.83
7.67
7.65
7.64
7.55
7.53
7.52
7.29
2.70
1.97
1.94
2.06
1.00
2.12
3.02
11.5
10.5
9.5
8.5
7.5
6.5
5.5
4.5
3.5
2.5
1.5
0.5
f1 (ppm)

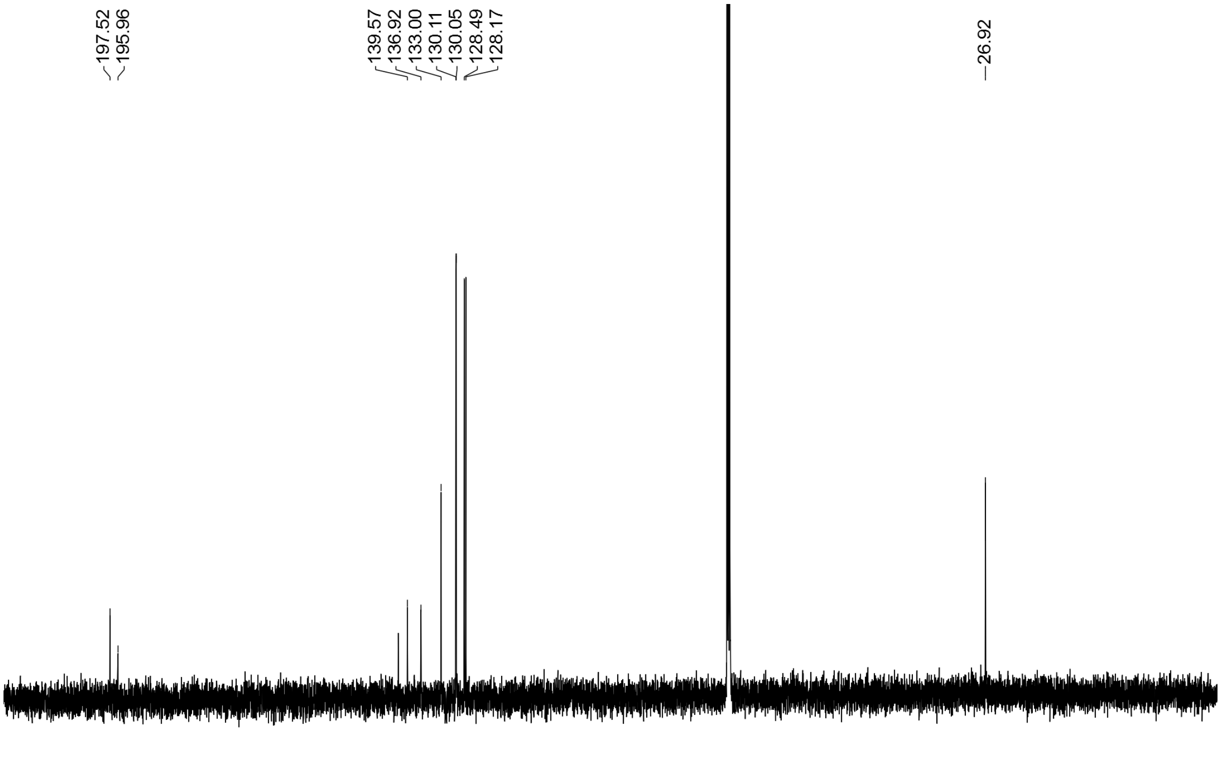
197.52
195.96
139.57
136.92
133.00
130.11
130.05
128.49
128.17
26.92
210
190
170
150
130
110
90
70
50
30
10
−10
f1 (ppm)

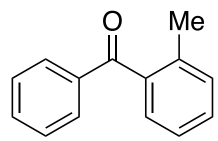
O
Me

3e

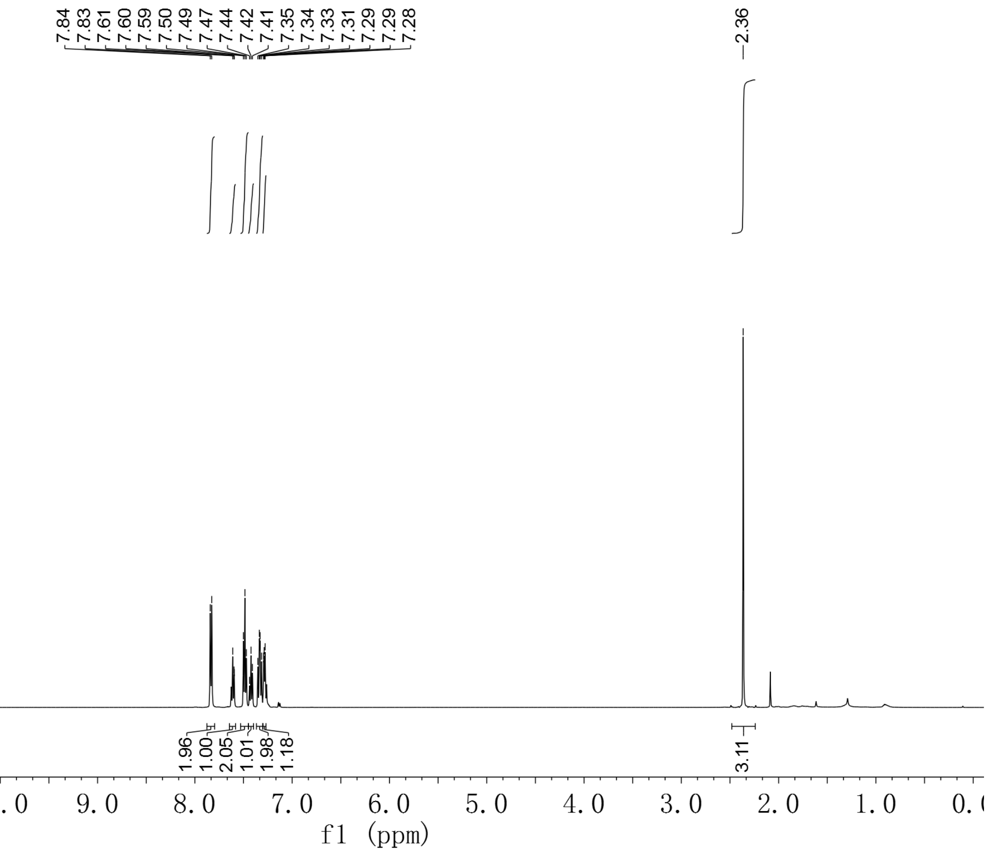
7.84
7.83
7.61
7.60
7.59
7.50
7.49
7.47
7.44
7.42
7.41
7.35
7.34
7.33
7.31
7.29
7.29
7.28
2.36
1.96
1.00
2.05
1.01
1.98
1.18
3.11
12.0 11.0 10.0 9.0 8.0 7.0 6.0 5.0 4.0 3.0 2.0 1.0
f1 (ppm)

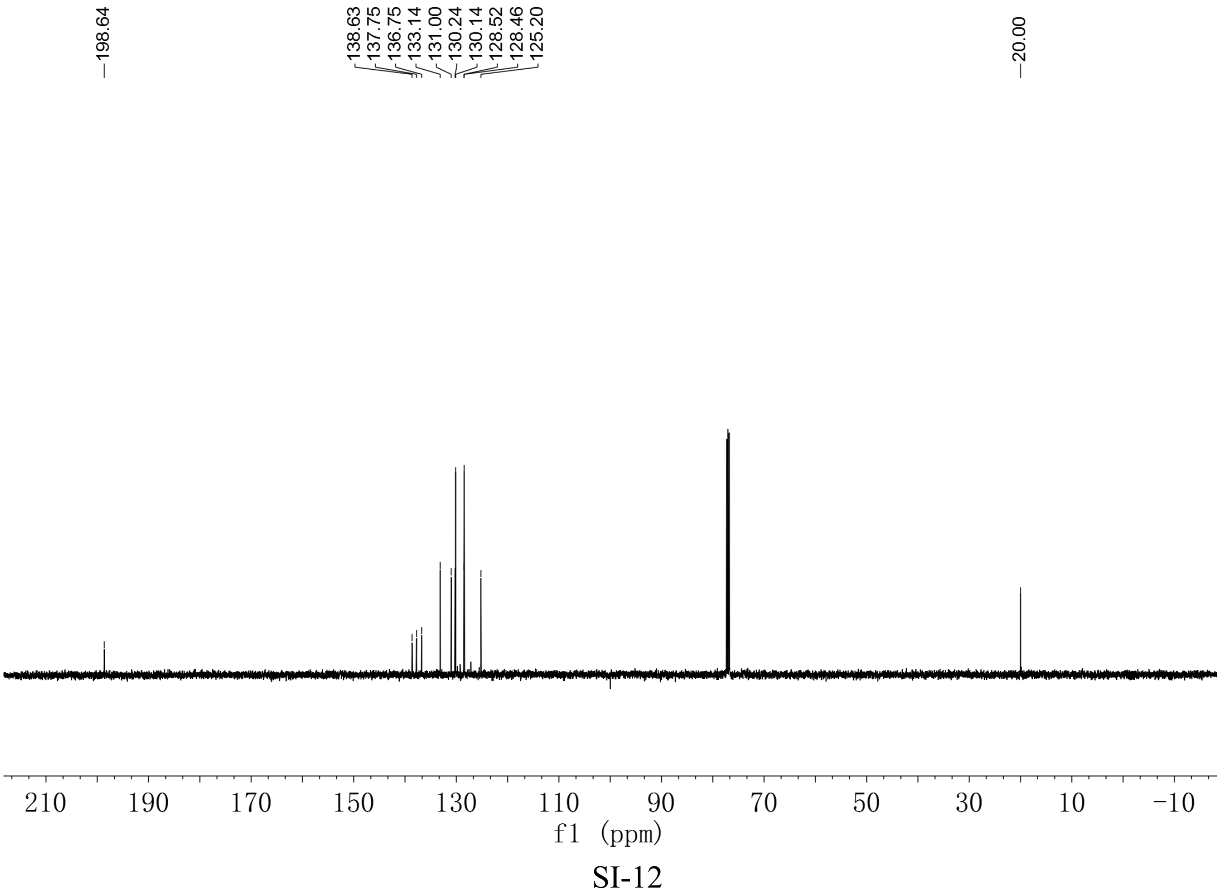
198.64
138.63
137.75
136.75
133.14
131.00
130.24
130.14
128.52
128.46
125.20
20.00
210 190 170 150 130 110 90 70 50 30 10 −10
f1 (ppm)

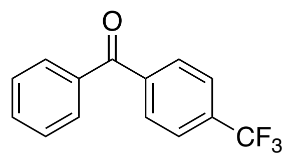
O
CF3

3f

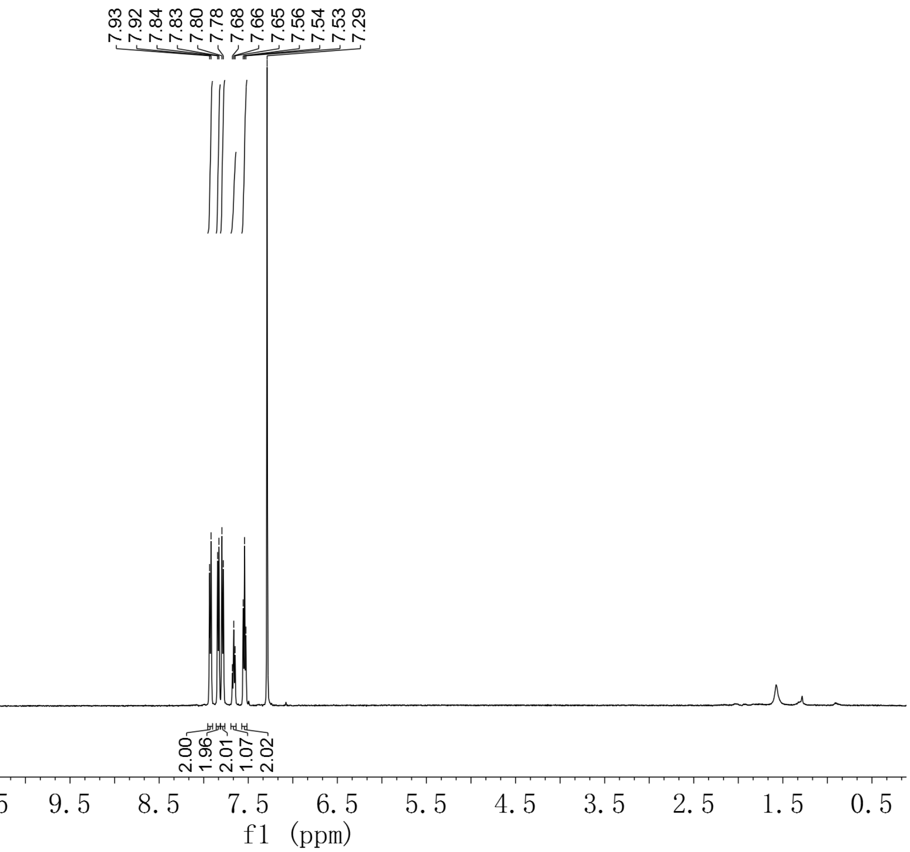
7.93
7.92
7.84
7.83
7.80
7.78
7.68
7.66
7.65
7.56
7.54
7.53
7.29
2.00
1.96
2.01
1.07
2.02
.3.5 12.5 11.5 10.5 9.5 8.5 7.5 6.5 5.5 4.5 3.5 2.5 1.5 0.5
f1 (ppm)

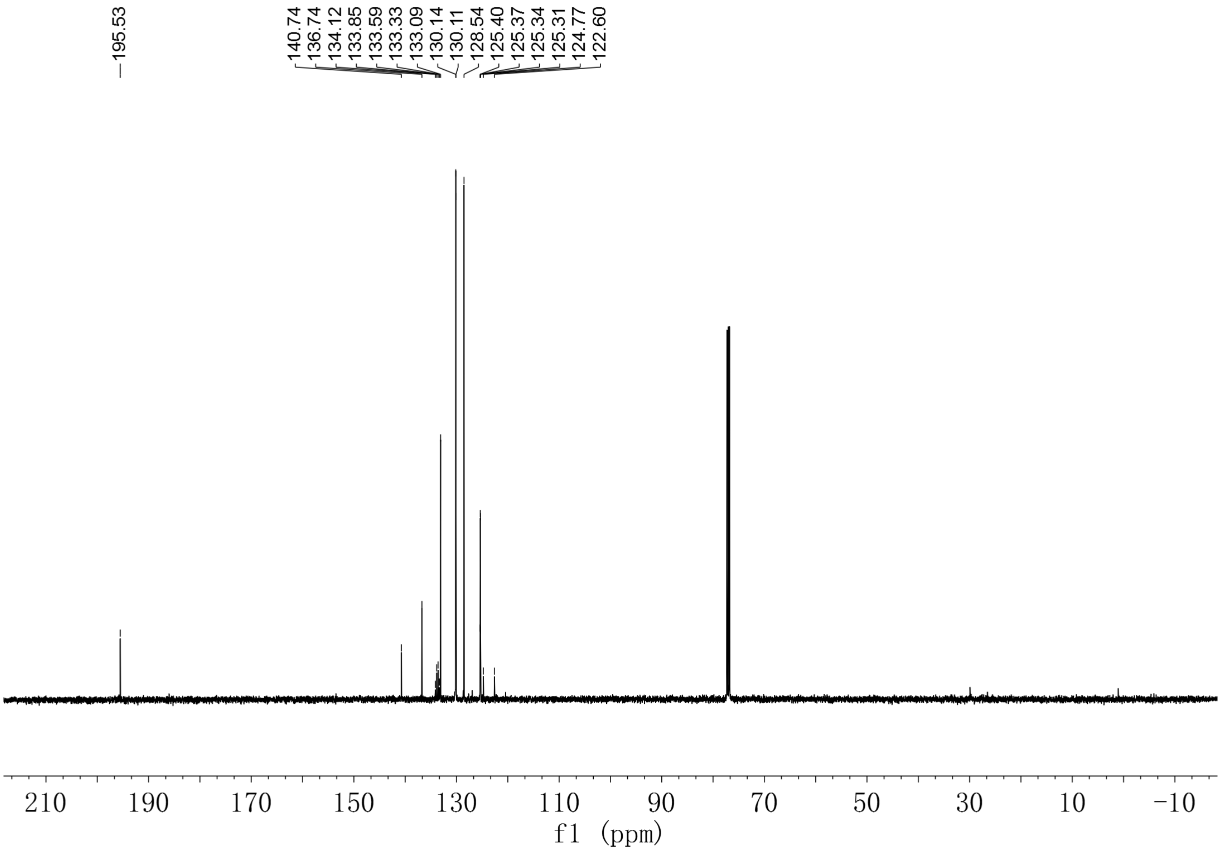
195.53
140.74
136.74
134.12
133.85
133.59
133.33
133.09
130.14
130.11
128.54
125.40
125.37
125.34
125.31
124.77
122.60
210 190 170 150 130 110 90 70 50 30 10 −10
f1 (ppm)

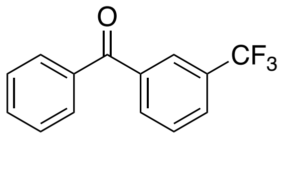
O
CF3

3g

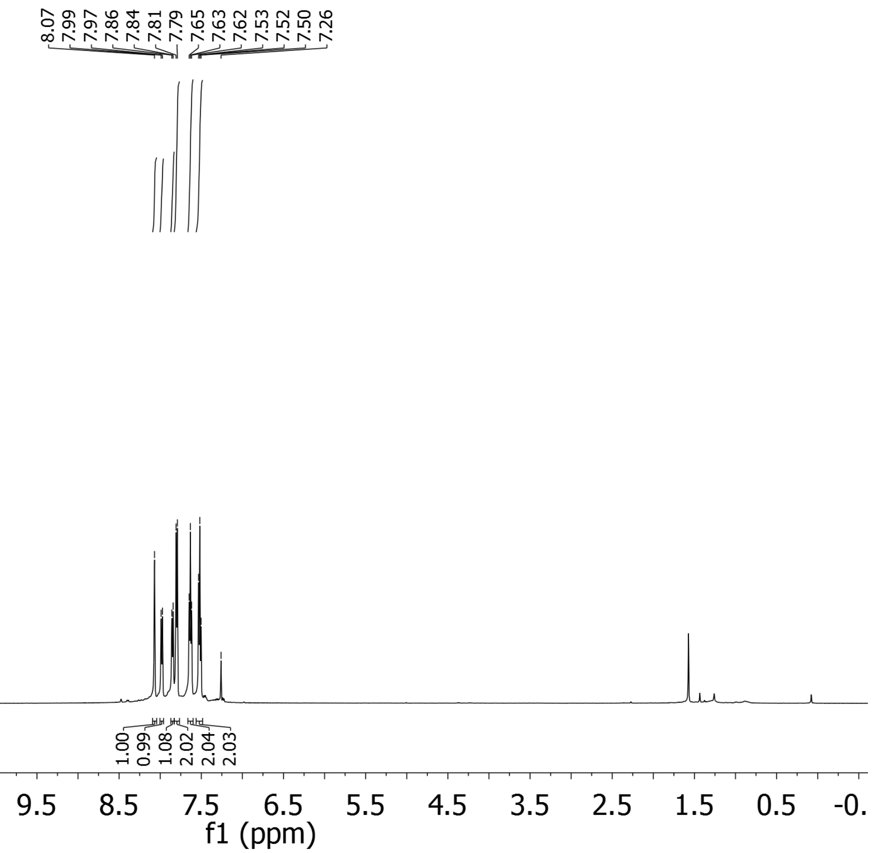
8.07
7.99
7.97
7.86
7.84
7.81
7.79
7.65
7.63
7.62
7.53
7.52
7.50
7.26
1.00
0.99
1.08
2.02
2.04
2.03
13.5 12.5 11.5 10.5 9.5 8.5 7.5 6.5 5.5 4.5 3.5 2.5 1.5 0.5 -0.
f1 (ppm)

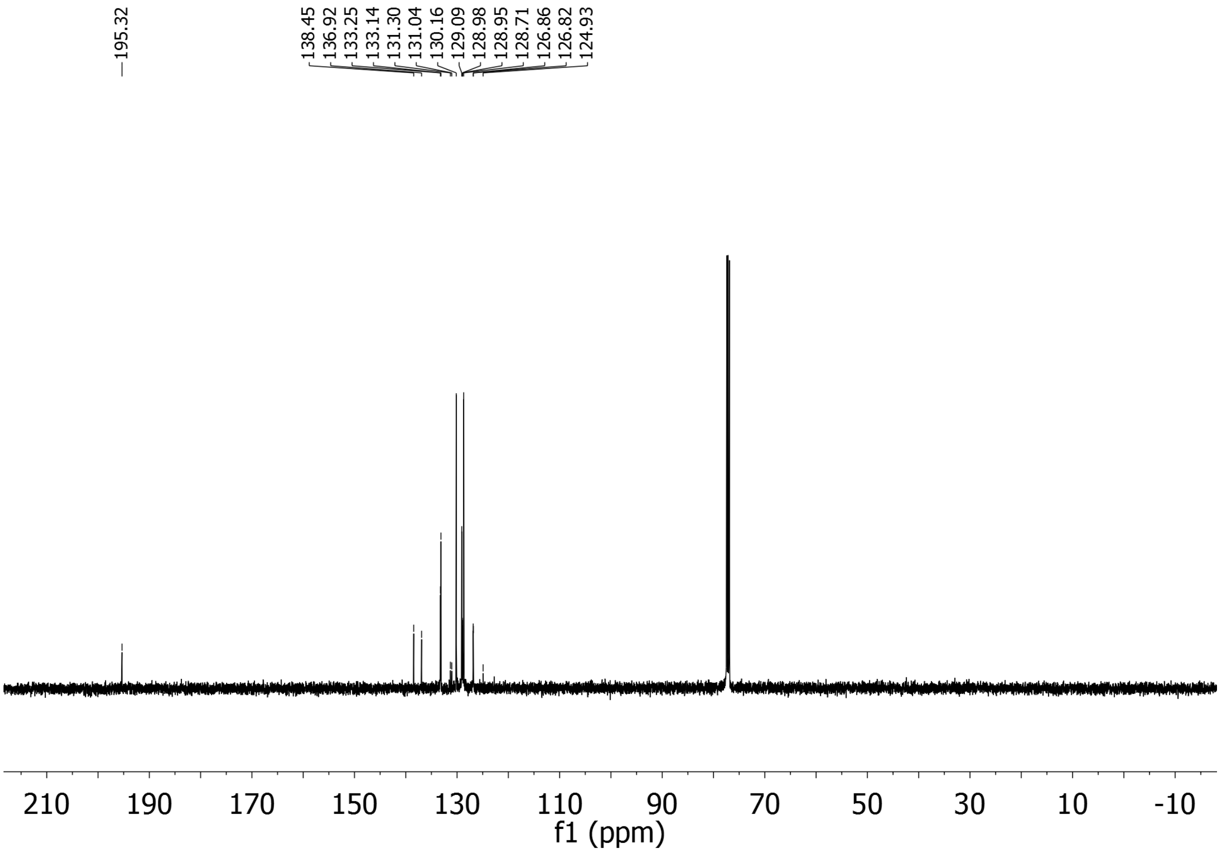
195.32
138.45
136.92
133.25
133.14
131.30
131.04
130.16
129.09
128.98
128.95
128.71
126.86
126.82
124.93
210 190 170 150 130 110 90 70 50 30 10 -10
f1 (ppm)

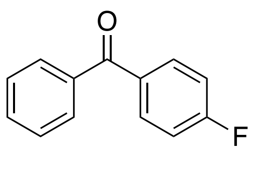
O
F

3h

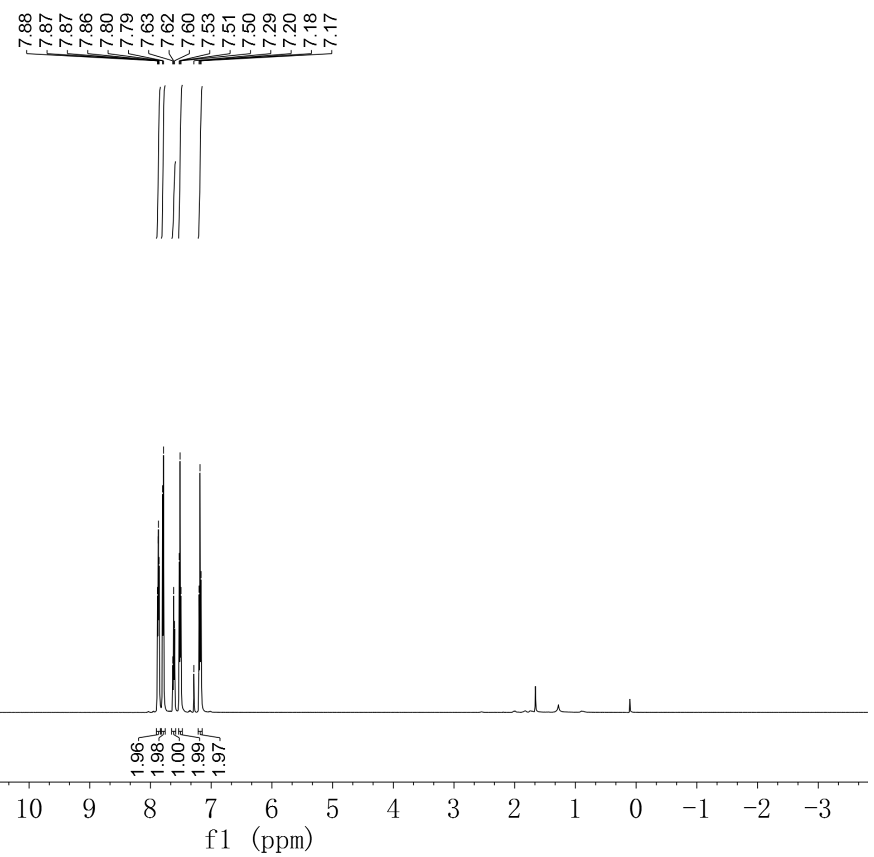
7.88
7.87
7.87
7.86
7.80
7.79
7.63
7.62
7.60
7.53
7.51
7.50
7.29
7.20
7.18
7.17
1.96
1.98
1.00
1.99
1.97
16 15 14 13 12 11 10 9 8 7 6 5 4 3 2 1 0 −1 −2 −3
f1 (ppm)

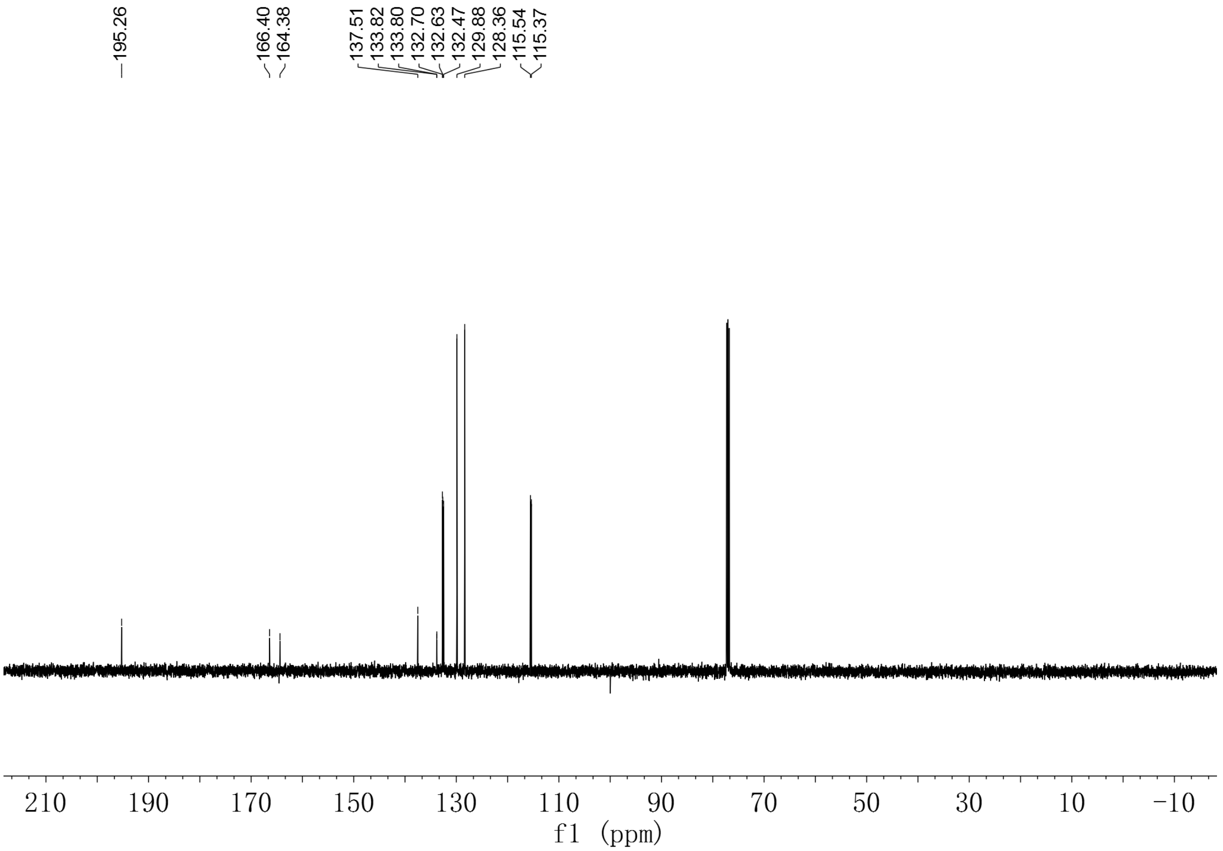
195.26
166.40
164.38
137.51
133.82
133.80
132.70
132.63
132.47
129.88
128.36
115.54
115.37
210 190 170 150 130 110 90 70 50 30 10 −10
f1 (ppm)

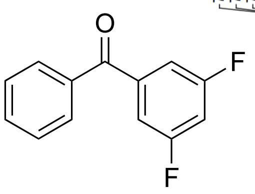
O
F
F

3i

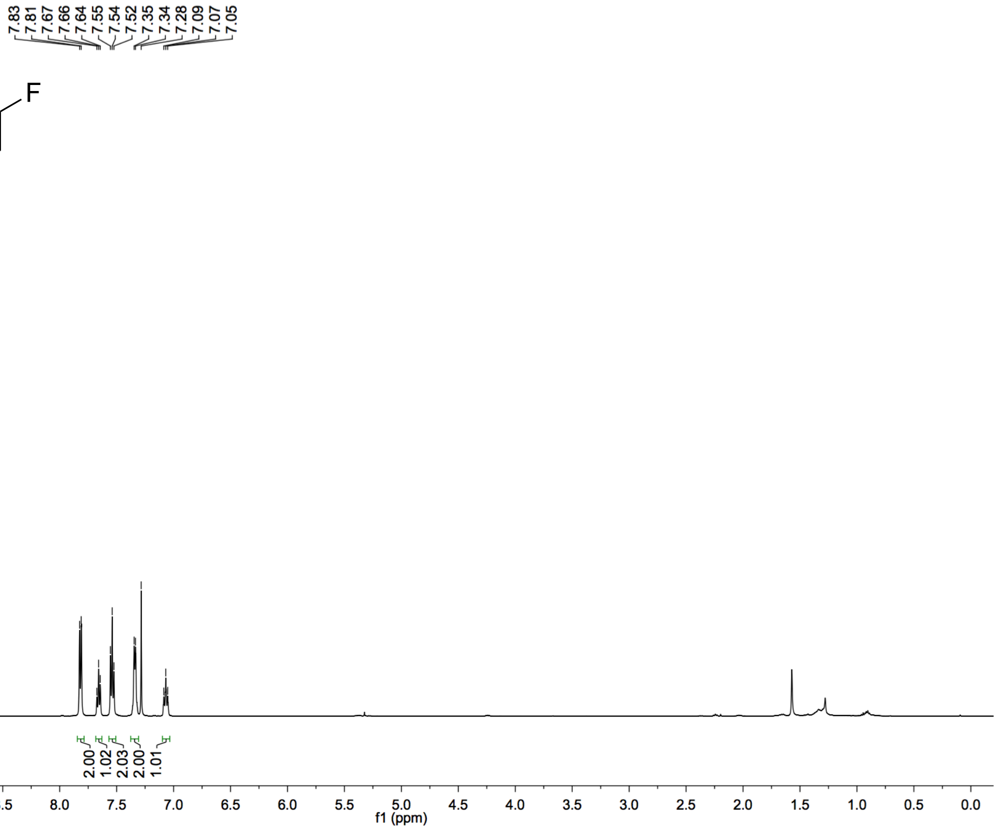
7.83
7.81
7.67
7.66
7.64
7.55
7.54
7.52
7.35
7.34
7.28
7.09
7.07
7.05
2.00
1.02
2.03
2.00
1.01
10.0 9.5 9.0 8.5 8.0 7.5 7.0 6.5 6.0 5.5 5.0 4.5 4.0 3.5 3.0 2.5 2.0 1.5 1.0 0.5 0.0
f1 (ppm)

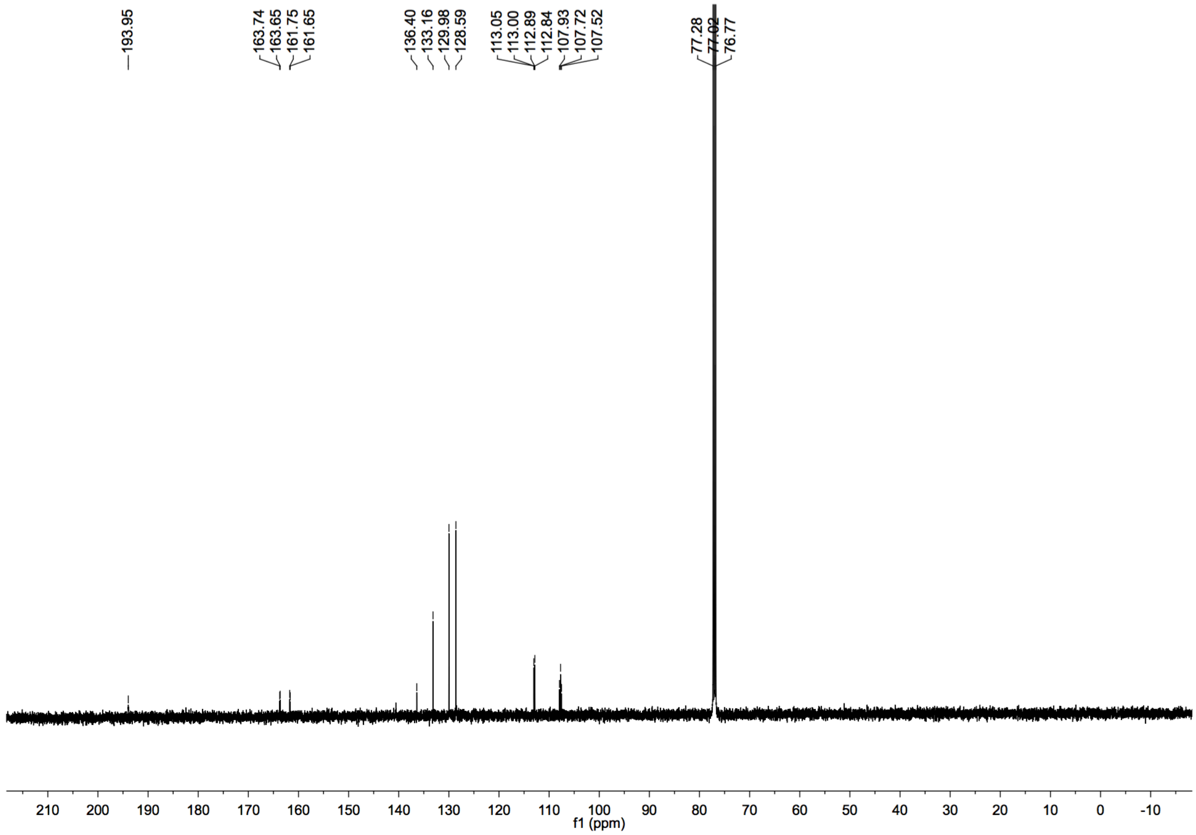
193.95
163.74
163.65
161.75
161.65
136.40
133.16
129.98
128.59
113.05
113.00
112.89
112.84
107.93
107.72
107.52
77.28
77.02
76.77
210 200 190 180 170 160 150 140 130 120 110 100 90 80 70 60 50 40 30 20 10 0 -10
f1 (ppm)

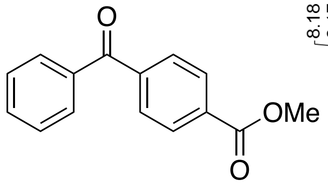
O
OMe
O

3j

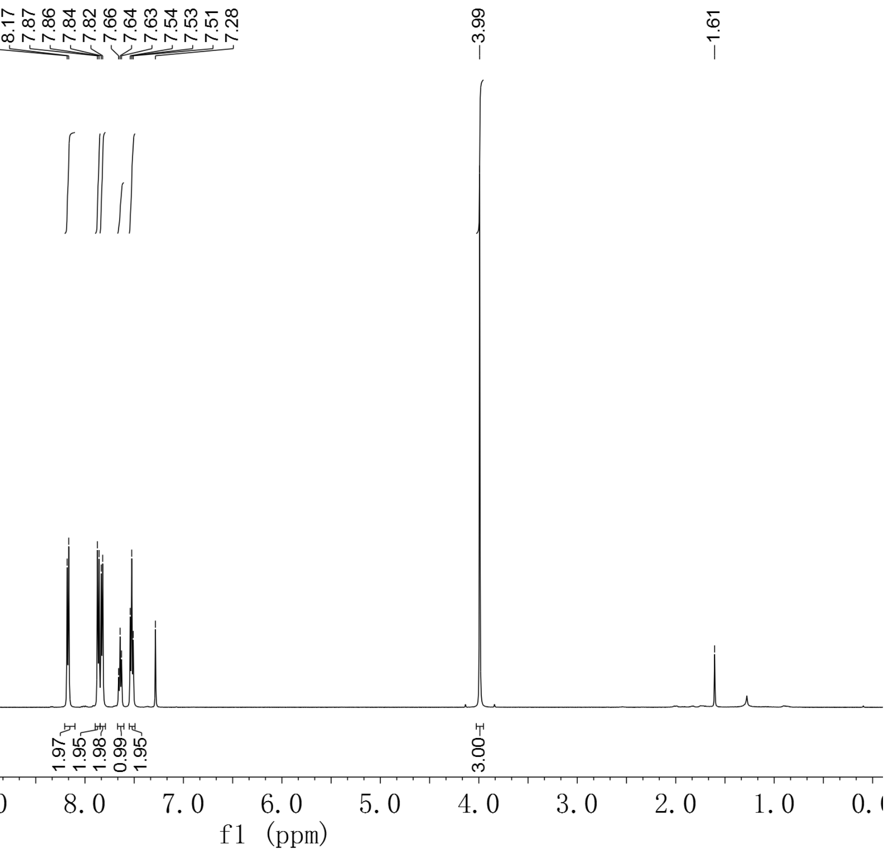
8.18
8.17
7.87
7.86
7.84
7.82
7.66
7.64
7.63
7.54
7.53
7.51
7.28
3.99
1.61
1.97
1.95
1.98
0.99
1.95
3.00
12.0
11.0
10.0
9.0
8.0
7.0
6.0
5.0
4.0
3.0
2.0
1.0
f1 (ppm)

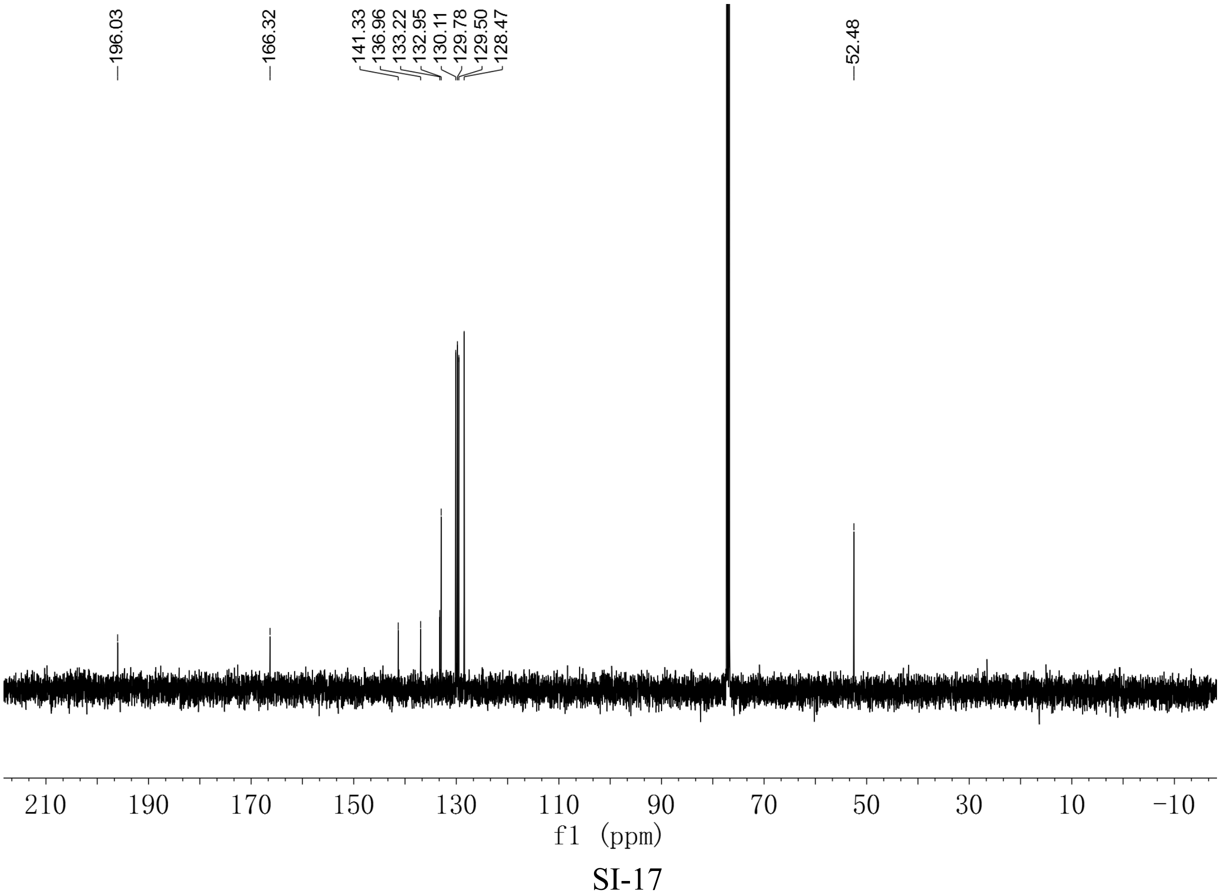
196.03
166.32
141.33
136.96
133.22
132.95
130.11
129.78
129.50
128.47
52.48
210
190
170
150
130
110
90
70
50
30
10
−10
f1 (ppm)

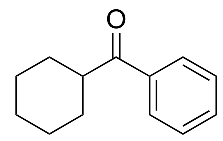

3k

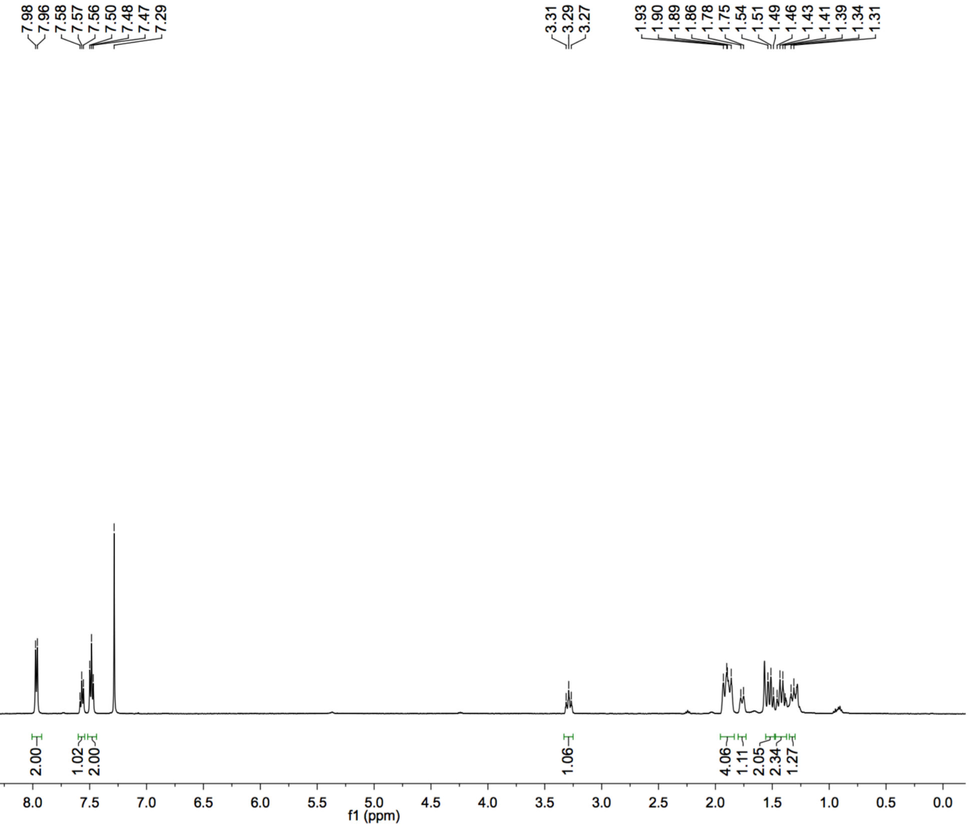

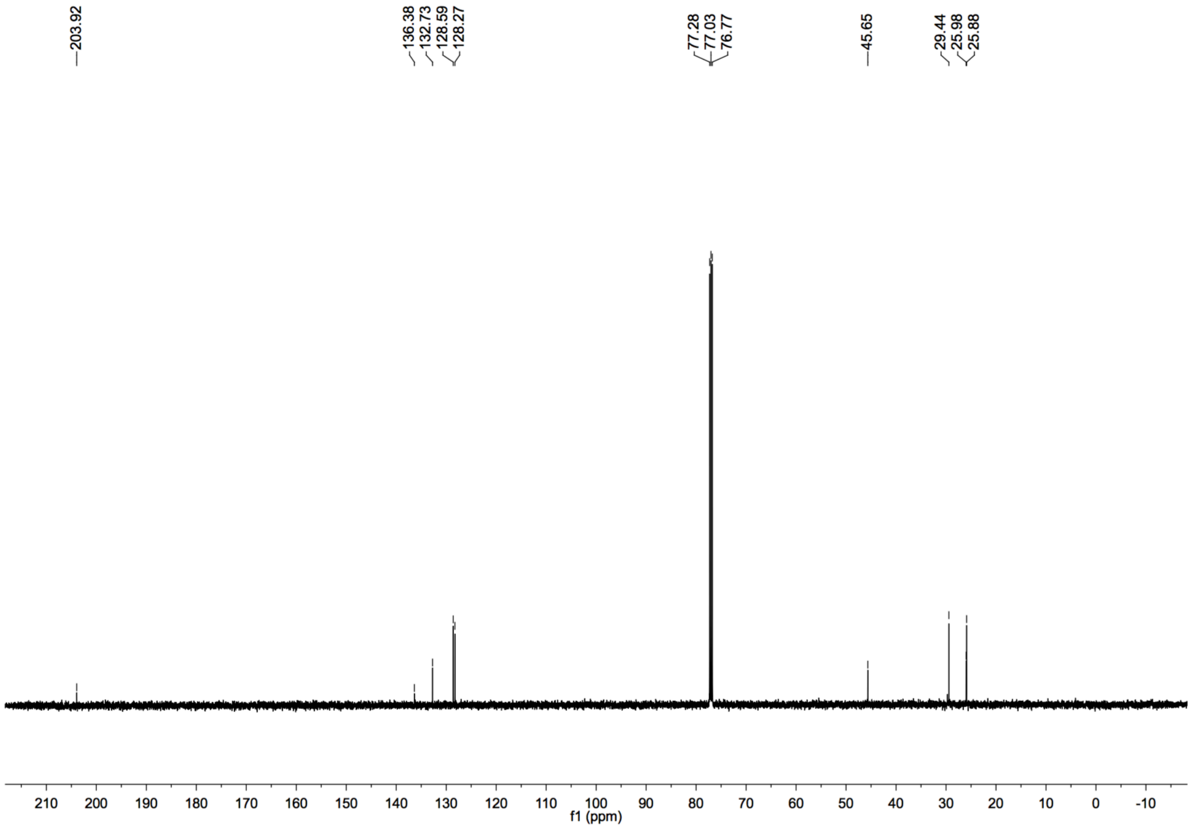

Supplement: Supplementary file 1 [file molecules-23-03134-s001.pdf]
